# Spray-Drying Microencapsulation of Grape Pomace Extracts with Alginate-Based Coatings and Bioaccessibility of Phenolic Compounds

**DOI:** 10.3390/gels11020130

**Published:** 2025-02-11

**Authors:** Josipa Martinović, Rita Ambrus, Mirela Planinić, Gabriela Perković, Gordana Šelo, Ana-Marija Klarić, Ana Bucić-Kojić

**Affiliations:** 1 Faculty of Food Technology Osijek, Josip Juraj Strossmayer University of Osijek, F. Kuhača 18, HR-31000 Osijek, Croatiamplanini@ptfos.hr (M.P.); gperkovic@ptfos.hr (G.P.); gselo@ptfos.hr (G.Š.); ana-marija.klaric@ptfos.hr (A.-M.K.); 2 Faculty of Pharmacy, Institute of Pharmaceutical Technology and Regulatory Affairs, University of Szeged, H-6720 Szeged, Hungary; ambrus.rita@szte.hu

**Keywords:** grape pomace, phenolic compounds, encapsulation, spray-drying, bioaccessibility

## Abstract

Spray-drying is a common technique for the microencapsulation of bioactive compounds, which is crucial for improving their stability and bioavailability. In this study, the encapsulation efficiency (*EE*), physicochemical properties and in vitro bioaccessibility of phenolic compounds from spray-dried encapsulated phenol-rich extracts of grape pomace, a winery waste, were evaluated. Sodium alginate alone (SA) or in a mixture with gum Arabic (SA-GA) or gelatin (SA-GEL) was used as a coating. SA-GEL achieved the highest *EE* (95.90–98.01%) and outperformed the intestinal release of phenolics by achieving a bioaccessibility index (*BI*) for total phenolic compounds of 37.8–96.2%. The release mechanism of phenolics from the microcapsules adhered to Fickian diffusion. Encapsulation significantly improved the *BI* of individual phenolics, with the highest *BI* values for gallocatechin gallate (2028.7%), epicatechin gallate (476.4%) and *o*-coumaric acid (464.2%) obtained from the SA-GEL microcapsules. Structural analysis confirmed amorphous matrices in all systems, which improved solubility and stability. These results suggest that encapsulation by spray-drying effectively protects phenolics during digestion and ensures efficient release in the intestine, which improves bioaccessibility. This study contributes to the understanding of biopolymer-based encapsulation systems, but also to the valorisation of grape pomace as a high-value functional ingredient in sustainable food processing.

## 1. Introduction

The increasing demand for sustainable practises in the food and dietary supplement industry has drawn attention to food industry residues such as grape pomace, which is a rich source of various bioactive compounds with a focus on phenolic compounds [1,2]. Phenolic compounds possess strong antioxidant, antimicrobial and antitumor properties, making them valuable for functional food formulations and health-related applications [3,4,5,6]. However, their inherent instability under processing, storage and digestion conditions poses a major challenge, so effective stabilization strategies are required to maintain their efficacy and improve their bioaccessibility and thus bioavailability [7,8]. Bioavailability refers to the proportion of a bioactive substance that is absorbed after ingestion and is available for biological activity at the target site. For a compound to reach this stage, however, it must first be bioaccessible, i.e., it must be released from the food matrix during digestion and be available for absorption in the gastrointestinal tract. Even if a compound is absorbed, its bioactivity, i.e., its ability to achieve a desired physiological or biochemical effect, depends on its stability, its interaction with metabolic pathways and its distribution in the body [9]. Phenolic compounds are challenging because of their complex chemical structure, but they are easily broken down in the acidic and enzymatic conditions found in the gastric phase of digestion, reducing their functional effects such as their antioxidant properties [10,11,12].

Encapsulation technologies such as spray-drying offer a promising solution to these challenges by embedding phenolic compounds in protective matrices [13]. This method not only stabilizes phenolics during processing, but also controls their release, which can improve bioavailability [14,15]. To optimize this process, the selection of suitable encapsulation materials is crucial, as their chemical properties significantly influence the protective function and release behaviour of the encapsulated compounds. In this study, three biopolymers—sodium alginate (SA), gum Arabic (GA) and gelatin (GEL)—were selected based on their complementary properties. SA, a natural polysaccharide derived from brown algae, forms a stable matrix due to its gelling ability, providing moderate protection for phenolic compounds [16]. However, its limited emulsifying properties can lead to lower encapsulation efficiency (*EE*), which is why combinations with other biopolymers are desirable [17]. GA, a plant-derived polysaccharide, complements sodium alginate by providing excellent emulsifying and film-forming properties, creating a robust encapsulation matrix that minimizes aggregation and can improve the retention of phenols during spray-drying [18]. GEL, a protein-based biopolymer, further improves the encapsulation system by exhibiting pH-sensitive swelling behaviour [19]. This property makes gelatin particularly effective for targeted release under intestinal conditions, where absorption predominantly occurs [8,20]. In addition, the helical protein structure of gelatin enables strong interactions with phenolic compounds, which improves their stability and controlled release [21].

The novelty of this study lies in its comprehensive evaluation of the *EE*, structural properties, in vitro release kinetics and bioaccessibility of phenolic compounds from encapsulated grape pomace extracts. To our knowledge, this is the first study to systematically investigate these aspects using SA, SA-GA and SA-GEL coatings for encapsulation using the spray-drying method. By studying the physicochemical properties and digestion and release profiles of these systems, we aim to identify the most effective formulation for the protection and release of phenolic compounds from grape pomace extracts.

## 2. Results and Discussion

### 2.1. Encapsulation Efficiency

**Figure 1:**
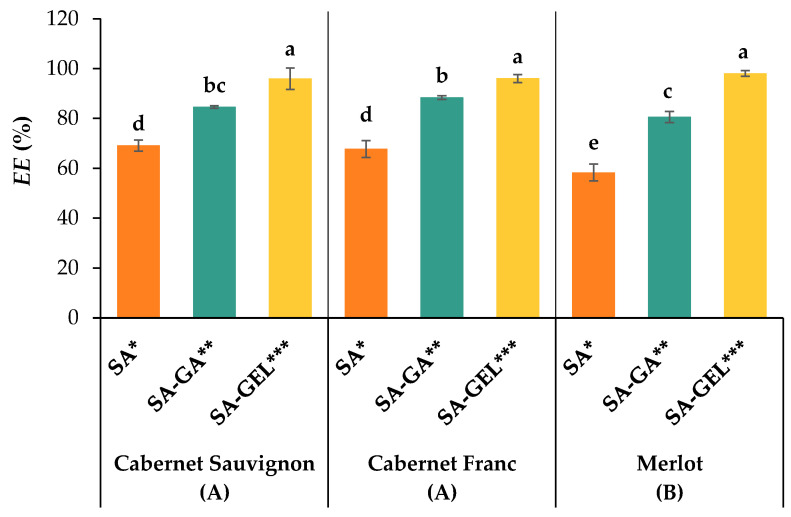
Encapsulation efficiency (*EE*, %) of phenol-rich grape pomace extracts using various coatings (SA—sodium alginate; SA-GA—combination of sodium alginate with gum Arabica; and SA-GEL—combination of sodium alginate with gelatin) (bar = mean; whisker = standard deviation). Different lower-case letters indicate statistically significant differences between the group (factors interaction), capital letters in brackets indicate differences between the grape pomace variety effects and different numbers of asterisks indicate differences between the coating effects according to Factorial ANOVA and the Tukey HSD post hoc test (*p* < 0.05).

Figure 1 shows the *EE* of spray-drying for the three grape pomace extracts and three coatings tested.

The lowest *EE* values were recorded when only SA was used, with results below 70% for all varieties. The limited effectiveness of SA in this context could be related to a lower ability to form stable protective layers, leading to greater losses of phenolic compounds during the spray-drying process. These findings support earlier research on other encapsulation techniques, such as ionic gelation and freeze-drying, and imply that SA must be mixed with other polymers to increase the effectiveness of bioactive component encapsulation [22,23].

According to Factorial ANOVA and the Tukey HSD post hoc test (*p* < 0.05), the type of grape pomace and the coating(s) applied have an effect on *EE*, and there is an interaction between these two factors (Figure 1). Coating had the greatest influence on *EE*, with 91.6% of the variance attributable to the variability of this factor, while the variability of the grape pomace variety factor and the interaction between the coating and grape pomace contributed 2.7% and 3.5% of the variance, respectively. For all samples, the highest *EE* (95.90–98.01%) was achieved with the SA-GEL coating. This excellent performance can be attributed to the improved protective properties and structural stability resulting from the SA-GEL coating. Song et al. [24] and Cortés-Morales et al. [25] described the synergistic effects of combining proteins and polysaccharides as coatings, leading to higher *EE* due to their complementary properties: while proteins stabilize and form films, polysaccharides form gels and provide protection. Conversely, the *EE* values of SA-GA ranged from 80.58% to 88.35% (Figure 1). GA is a highly branched polysaccharide that improves emulsion stability and provides moderate protection from environmental conditions [26,27]. Its *EE* was lower than that of the SA-GEL combination, most likely due to fact that its gel network was weaker. Nevertheless, the SA-GA coating significantly improved the *EE* compared to SA alone, suggesting that it could be a promising encapsulation coating combination in situations where protein-based materials are undesirable. Microcapsules containing an extract of Merlot grape pomace had the lowest *EE* and were statistically different from CS and CF.

### 2.2. Physicochemical Properties of Microcapsules

Microcapsules were produced by spray-drying grape pomace extract, rich in phenolic compounds, with a variety of alginate-based coatings. A physicochemical characterization of the microcapsules was performed by morphology analysis, X-ray powder diffraction (XRPD) and differential scanning calorimetry (DSC).

#### 2.2.1. Scanning Electron Microscopy of Microcapsules

**Figure 2:**
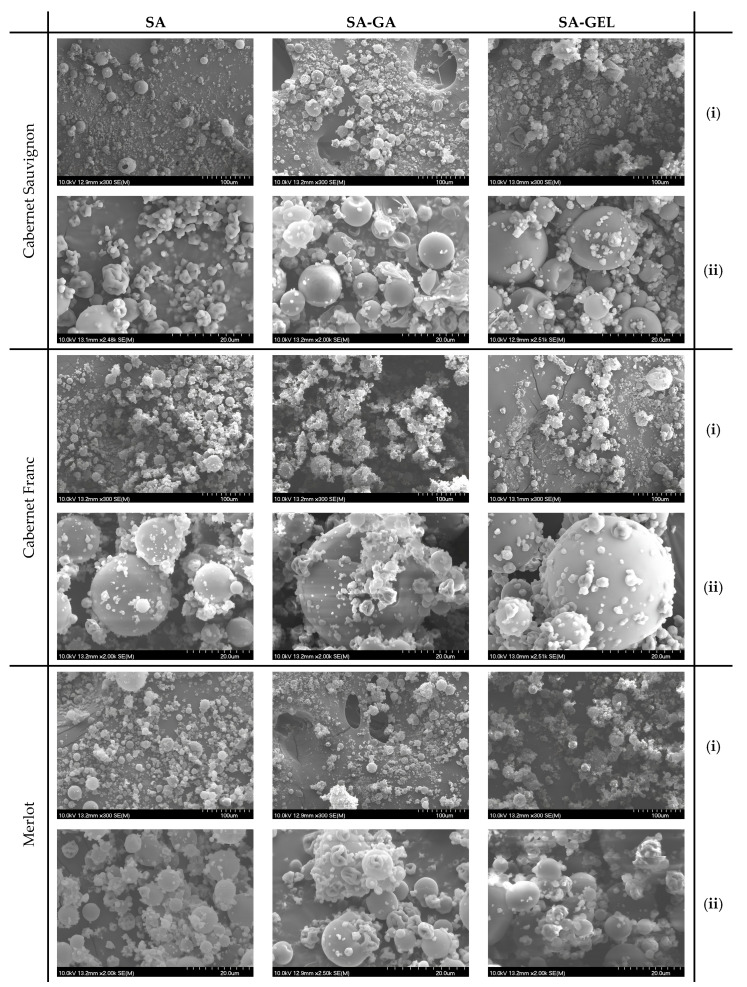
SEM images of microcapsules prepared with different coatings containing extracts from different grape pomace extracts. The images are at a scale of 100 µm (**i**) and 20 µm (**ii**).

The morphology of the microcapsules containing different pomace extracts was explored by scanning electron microscopy (SEM). SEM images (Figure 2) provide an insight into the structural properties of the microcapsules.

When spray-dried, the SA microcapsules show a mostly spherical shape, and their closer inspection discloses size variation and some irregularities, probably due to the aggregation of the particles during the drying process. The majority of the particles were generally smooth in surface texture with slight indentations or wrinkles, especially observed in Cabernet Sauvignon SA microcapsules (Figure 2 SA(ii)), most likely due to moisture loss during encapsulation or process conditions. Further, some coalescence can be observed between the SA microcapsules of the Cabernet Franc and Merlot extracts (Figure 2), indicating also that the coating of SA has a limited effectiveness in preventing fusion. This phenomenon has also been observed by other studies, such as by Tonon et al. [28] and Botrel et al. [29]. When SA was combined with GA, Cabernet Sauvignon SA-GA microcapsules resulted in a more uniform shape. The surface of these particles could be more rugged (as seen for the Merlot microcapsules in Figure 2 SA-GA(ii)), owed to the properties of GA, known for its emulsifying and film-forming abilities that enhance particle stability, reducing coalescence. The combination SA-GA provides an encapsulation matrix that is much more cohesive and hence allows better separation between particles; overall, it allows more effective encapsulation. In contrast, SA combined with gelatin, SA-GEL, gives microcapsules that maintain a generally spherical form apart from some irregularities seen in the Cabernet Sauvignon microcapsules (Figure 2 SA-GEL(ii)). These irregularities could be due to the lower rigidity of GEL compared to GA, leading to increased surface variability. Nevertheless, GEL may offer additional benefits such as an improvement in bioavailability and the controlled release of the encapsulated substances [30].

Table 1 illustrates the variations in the particle size distribution of the spray-dried microcapsules.

Cabernet Sauvignon microcapsules coated with SA showed the widest size range (1.97–11.98 μm) and the largest mean diameter (3.96 μm). A wide size distribution is indicated by the D10, D50 and D90 values (2.08 μm, 3.03 μm and 6.95 μm, respectively). On the other hand, SA-GA- and SA-GEL-coated microcapsules displayed smaller mean diameters of 2.61 μm and 2.62 μm, respectively. The size distributions produced by both the SA-GA and SA-GEL combinations were more consistent and had smaller ranges (1.19–10.69 μm for SA-GA and 1.18–12.02 μm for SA-GEL). The same pattern was seen in Cabernet Franc microcapsules. The SA microcapsules had the largest mean diameter of 4.29 μm and the broadest size range (1.97–11.90 μm), with D10, D50 and D90 values of 2.10 μm, 3.46 μm and 7.37 μm, respectively. The mean diameters of SA-GA (2.59 μm) and SA-GEL (2.58 μm) were lower, with smaller size ranges of 1.18–10.08 μm and 1.18–12.17 μm, respectively. Out of all the grape pomace varieties used, the Merlot microcapsules were the smallest ones. The SA microcapsules had an average diameter of 2.82 μm and ranged in size from 1.19 to 12.23 μm. The D10, D50 and D90 values (1.24 μm, 1.95 μm and 5.81 μm, respectively) showed a broad range of sizes. On the other hand, the microcapsules coated with SA-GA and SA-GEL showed narrower ranges (1.18–11.56 μm for SA-GA and 1.18–12.31 μm for SA-GEL), with smaller mean diameters of 2.53 μm and 2.38 μm, respectively. Interestingly, SA-GEL-coated particles had the highest homogeneity and the narrowest distribution (D10–D90: 1.18–4.57 μm).

This particle size analysis highlights consistent trends across grape varieties and coatings. SA-GA and SA-GEL coatings produced smaller, more homogeneous particles, whereas SA coatings produced the biggest particles with a broader size distribution. Information on particle size distribution and diameter emphasizes how crucial it is to choose coating materials based on certain encapsulation and release specifications.

#### 2.2.2. Thermal and Structural Analysis of Coatings and Microcapsules

The various coatings used (SA-0, GA-0, GEL-0), as well as the spray-dried microcapsules produced, were analyzed for their crystalline and amorphous state using XRPD and DSC.

**Figure 3:**
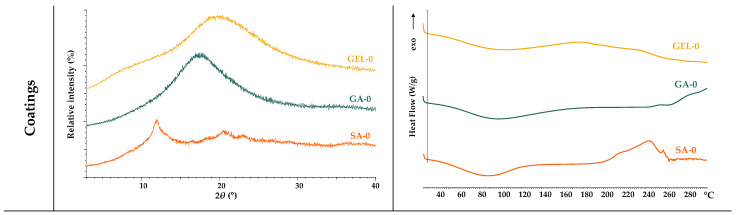

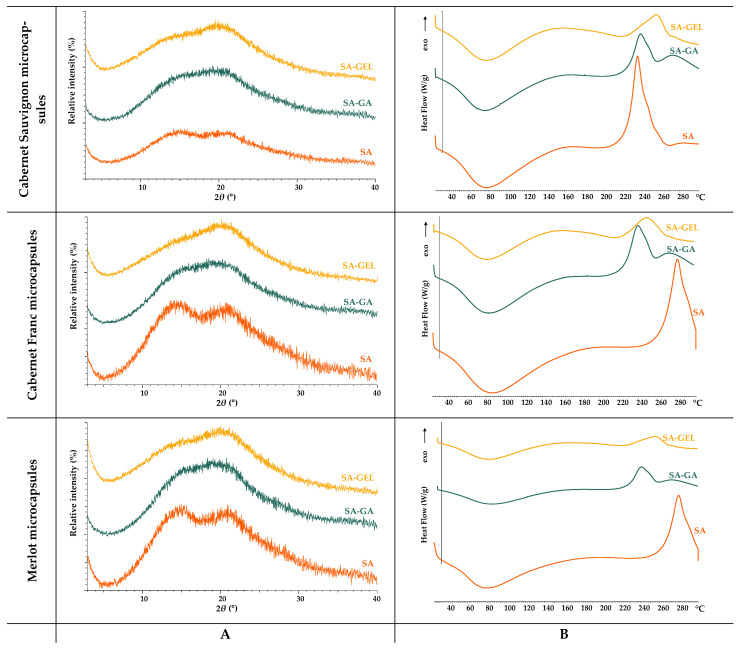
XPRD diffractograms (**A**) and DSC thermograms (**B**) of the coatings and microcapsules containing the tested extracts.

The XRPD analysis revealed that all coatings investigated were amorphous, as shown in Figure 3A. While SA-0 shows a clearly defined peak, GA-0 and GEL-0 show broader and less pronounced peaks, all of which are characteristic of an amorphous state. As for the thermal behaviour observed using DSC, both SA-0 and GA-0 exhibit a broad endothermic peak in the low-to-medium temperature range (60–120 °C), which is related to dehydration. On the other hand, consistent with the findings of Guan and Zhong [31], GA-0 demonstrates higher thermal stability as its thermogram does not display exothermic event, implying it can endure higher temperatures without undergoing degradation. In contrast, the observed degradation of SA-0 at high temperatures, specifically at 240–260 °C (Figure 3B), aligns with the results of Barra et al. [32]. The water loss of GEL-0 occurs in a slightly broader temperature range (50–176 °C) and is followed by a pronounced endothermic peak between 230 °C and 250 °C, which represents the denaturation or melting of the helical structure. Dai et al. [33] published that this transition reflects the structural stability and unique ordering attributed to the helical conformation. From the DSC results, it can be seen that the coatings tested in the current study have an amorphous structure, which confirms the XRPD results obtained above.

The microcapsules coated with SA-GA and SA-GEL show more similar diffraction patterns in all the samples than those coated with SA. The peak intensities of all microcapsules vary slightly between 10° and 30°, which is probably due to slight interactions between the extract and the encapsulation matrix. The DSC thermograms (Figure 3B) show a dehydration peak, as seen in the thermograms of the coatings used, followed by the thermal transitions. Sharp transitions at higher temperatures could indicate interactions between the coatings and the phenolic compounds from the grape pomace extracts. It appears that simpler polysaccharide coatings in the microcapsules such as SA have sharper transitions in DSC due to more thermal degradation, while broader transitions are observed in the SA-GA and SA-GEL microcapsules, with a slight increase in thermal stability compared to the SA microcapsules. Barra et al. [32] interpreted this as evidence of either slow degradation or more complex interactions with encapsulated material.

### 2.3. Enzyme-Free Release Study of Phenolic Compounds

**Figure 4:**
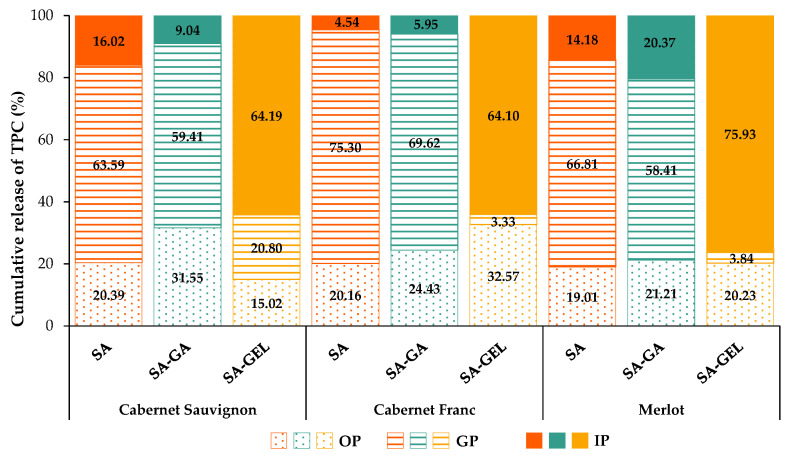
The percentage of total phenolic compound (TPC) release from microcapsules produced with different coatings containing the tested extracts at the end of each gastrointestinal phase.

The release of total phenolic compounds (TPCs) from the microcapsules under conditions mimicking the human gastrointestinal system through three digestive phases, an oral (OP), gastric (GP) and intestinal (IP) phase, using appropriate electrolyte solutions without digestive enzymes is shown in Figure S1 and Figure 4. The monitoring of TPC release under the above conditions lasted a total of 243 min, with OP lasting 3 min, after which GP was continued for 120 min and IP for a further 120 min.

The cumulative TPC release profile of the microcapsules containing grape pomace extracts shows a clear release pattern across all digestive phases, as shown in Figure S1 and Figure 4. For microcapsules coated only with SA, TPC release starts in the OP with values between 10.42 and 13.35 mg_GAE_/g_MC_, indicating a rapid initial release (Figure S1). This initial release could be due to phenolics absorbed at the surface, which are not well incorporated into the microcapsule matrix and are therefore the first to be released. During the GP, SA microcapsules show an increase, reaching cumulative release values between 18.31 and 42.93 mg_GAE_/g_MC_ for Cabernet Sauvignon, 25.31 and 63.22 mg_GAE_/g_MC_ for Cabernet Franc and 23.16 and 55.73 mg_GAE_/g_MC_ for Merlot microcapsules (Figure S1). However, release into the IP is minimal, with the cumulative TPCs increasing only slightly to about 51.12–66.23 mg_GAE_/g_MC_, suggesting a moderate role of SA in protecting TPCs in early digestion. This is best illustrated in Figure 4, where it can be seen that the highest release of TPCs occurs in the GP (63.59–75.30%), where the coating should provide the greatest protection.

For all SA-GA microcapsules, the release in the OP is similar, with cumulative TPC values ranging from 11.00 to 13.61 mg_GAE_/g_MC_ (Figure S1). In the GP, the release is more substantial, reaching a peak value of 38.11–52.39 mg_GAE_/g_MC_ (Figure S1), or 58.41–69.62% (Figure 4). This moderate release then continues in the IP, where the TPC values at the end of the IP are 38.93–55.71 mg_GAE_/g_MC_. Both SA and SA-GA follow a comparable release trend, but SA-GA provides slightly more protection, probably due to the additional stabilizing effects of gum Arabic. This makes SA-GA more effective in maintaining TPC stability in the early stages of digestion, especially compared to SA.

In contrast, the SA-GEL microcapsules show a very phase-specific release pattern, which distinguishes them from the SA and SA-GA coatings. During the OP, the release of TPCs is minimal, with values between 4.74 and 12.89 mg_GAE_/g_MC_ (Figure S1). This initial retention of TPCs is likely related to the minimal presence of surface phenolic compounds (SPCs) in the SA-GEL microcapsules compared to the SA and SA-GA microcapsules, which is consistent with the observed lower initial release in the oral environment. As digestion progresses to the GP, TPC release from the SA-GEL microcapsules increases, reaching values between 7.33 and 11.29 mg_GAE_/g_MC_ for Cabernet Sauvignon, 10.05 and 14.21 mg_GAE_/g_MC_ for Cabernet Franc and 5.38 and 8.36 mg_GAE_/g_MC_ for Merlot microcapsules (Figure S1), corresponding to 3.33–20.80% of the TPCs (Figure 4). The release of TPCs is sustained in the GP, suggesting that the SA-GEL combination retains the protective capacity for TPCs in the stomach as in the mouth, preventing rapid degradation or release and allowing the gradual diffusion of TPCs from the microcapsules. Upon the transition to the IP, the SA-GEL microcapsules show a significant increase in TPC release, with cumulative values increasing to 31.53–39.58 mg_GAE_/g_MC_ (Figure S1), i.e., 64.10–75.93% (Figure 4). This significant release in the IP shows that the SA-GEL coating is highly responsive to the pH conditions prevailing in the intestine. The gelatin in the coating likely swells or partially dissolves under the neutral to slightly basic pH in the intestinal phase, significantly increasing the diffusion and release of TPCs. This behaviour reflects the pH-sensitive properties of gelatin, which can swell and become more permeable under intestinal conditions [34], facilitating the targeted release of TPCs at the preferred absorption site.

The release kinetics of TPCs from microcapsules were evaluated using different mathematical models: the first order model, Higuchi model, Hixson–Crowell model and Korsmeyer–Peppas model (Figure S2). Table 2 summarizes the model parameters (*k* values and the diffusion exponent *n* for the Korsmeyer–Peppas model) and the statistical criteria used to evaluate the suitability of the model for data approximation. Of the statistical parameters, the R^2^_adj_ adjusted coefficient of determination was the leading parameter in selecting the most appropriate model.

Table 2 shows that the Korsmeyer–Peppas empirical model best describes the release kinetics of TPCs from the SA and SA-GA microcapsules of the Cabernet Sauvignon and Merlot extracts (R^2^_adj_ > 0.91), as it is flexible enough to describe complex release behaviour as described in numerous articles such as Korsmeyer et al. [35], Costa and Sousa Lobo [36] and Siepmann and Siepmann [37]. However, considering the graphical representation (Figure S2), the fractional release of TPCs from these microcapsules is better described by a first order model. Therefore, since the first order model is a mechanistic model, it reflects the observed release kinetics more accurately. Nevertheless, the values of the diffusion exponent *n* obtained for SA and SA-GA microcapsules (≤0.45) indicate that Fick’s diffusion is the primary release mechanism (Table 2). For Cabernet Franc, the first order model yielded the highest R^2^_adj_ of 0.937 for SA and 0.916 for SA-GA microcapsules, which is consistent with the plot in Figure S2 and generally indicates that this model characterizes the release kinetics for all SA and SA-GA microcapsules well.

It can be seen from Figure S2 that the release profile of the SA-GEL microcapsules was different from that of the SA and SA-GA microcapsules and cannot be fully described by any of the tested models, although the Higuchi model provides the best statistical parameters to approximate the data (R^2^_adj_ between 0.801 and 0.852) (Table 2). In this case, three phases of phenol release are observed, with the first phase showing a tendency to increase and then decrease, the second showing a steady-state release in the GP and the third a gradual increase in the small intestine. This trend indicates that the release of phenolic compounds from the SA-GEL microcapsules was most sensitive to the pH environment in which the release took place.

### 2.4. Bioaccessibility of Phenolic Compounds

Simulated digestion in vitro with digestive enzymes mimicking the human upper digestive system was performed with non-encapsulated grape pomace extracts of Cabernet Sauvignon (CS), Cabernet Franc (CF) and Merlot (M), i.e., control samples (C_CS_; C_CF_; C_M_), as well as with microencapsulated extracts (SA; SA-GA; SA-GEL). To assess the phenolic compounds’ bioaccessibility, the digestion was carried out in the OP, GP, and IP. The content of the individual phenolic compounds of the control samples before the simulated digestion in vitro is shown in Tables S1–S3. The detailed chemical composition of these extracts was analyzed in a previously published study by Martinović et al. [23].

**Figure 5:**
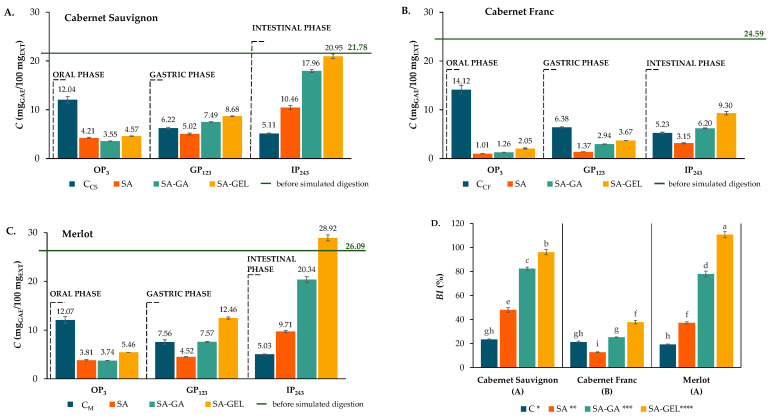
TPCs before (green line) and after each phase of simulated gastrointestinal digestion (bar = mean; whisker = standard deviation) of non-encapsulated (control, C) and microencapsulated extracts (SA; SA-GA; SA-GEL) of different grape pomaces ((**A**)—Cabernet Sauvignon; (**B**)—Cabernet Franc; (**C**)—Merlot) and bioaccessibility index (*BI*) of TPCs—(**D**). [Different lower-case letters indicate statistically significant differences between group (factors interaction), capital letters in brackets indicate differences between grape variety effects and different numbers of asterisks indicate differences between coating effects according to Factorial ANOVA and Tukey HSD post hoc test (*p* < 0.05)].

**Figure 6:**
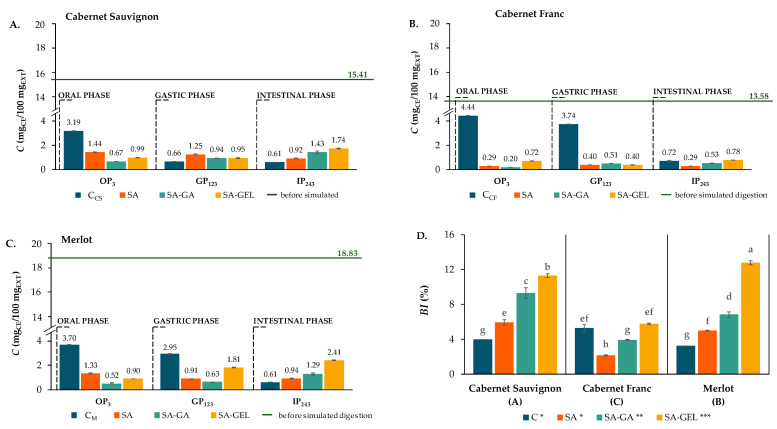
Total flavonoid content (TFC) before (green line) and after each phase of simulated gastrointestinal digestion (bar = mean; whisker = standard deviation) of non-encapsulated (control, C) and microencapsulated extracts of different grape pomaces ((**A**)—Cabernet Sauvignon; (**B**)—Cabernet Franc; (**C**)—Merlot) and *BI* of TPCs—(**D**). [Different lower-case letters indicate statistically significant differences between group (factors interaction), capital letters in brackets indicate differences between grape variety effects and different numbers of asterisks indicate differences between coating effects according to Factorial ANOVA and Tukey HSD post hoc test (*p* < 0.05)].

**Figure 7:**
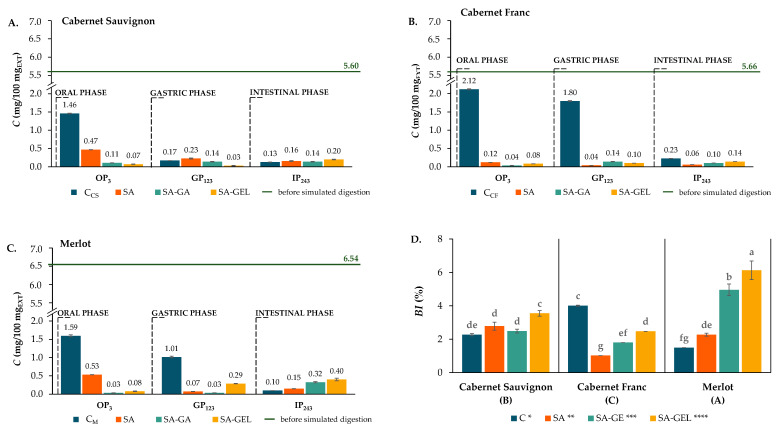
Total extractable proanthocyanidin content (TPA) before (green line) and after each phase of simulated gastrointestinal digestion (bar = mean; whisker = standard deviation) of non-encapsulated (control, C) and microencapsulated extracts of different grape pomaces ((**A**)—Cabernet Sauvignon; (**B**)—Cabernet Franc; (**C**)—Merlot) and *BI* of TPA—(**D**). [Different lower-case letters indicate statistically significant differences between group (factors interaction) capital letters in brackets indicate differences between grape variety effects and different numbers of asterisks indicate differences between coating effects according to Factorial ANOVA and Tukey HSD post hoc test (*p* < 0.05)].

During the simulated digestion, samples were taken at a certain period of time (e.g., OP_3_, GP_123_ and IP_243_; where the numerical index indicates the duration of digestion in minutes). These samples were analyzed for TPCs, total flavonoid content (TFC) and total proanthocyanidin content (TPA), as well as for the content of individual phenolic compounds such as phenolic acids, stilbenes, flavanols, flavonols and anthocyanins. The aim was to evaluate the effects of the coatings used on the release and bioaccessibility index (*BI*) of phenolics encapsulated by spray-drying (Figure 5D, Figure 6D and Figure 7D). Equation (3) was used to determine the *BI* based on the quantity of phenolic compounds in the tested sample before digestion (BD) and after the IP.

#### 2.4.1. Total Phenolics, Total Flavonoids and Total Proanthocyanidins

The TPCs, TFC and TPA released from the control samples and the microcapsules in the enzyme-containing digestion fluids after each digestion phase, as well as the *BI* of these samples, are shown in Figure 5, Figure 6 and Figure 7.

Encapsulated extracts in the OP showed a noticeably lower TPC release than the control samples for each variety (Figure 5A–C). For instance, 14.12 mg_GAE_/100 mg_EXT_ was released from the control C_CF_, while only 1.01 to 2.05 mg_GAE_/100 mg_EXT_ was released from the encapsulated Cabernet Franc samples, which were also the lowest concentrations of TPCs released from all the microcapsules (Figure 5B). Merlot and Cabernet Sauvignon showed a similar release trend (Figure 5A,C). In line with De Vos et al. [38], this decrease in release is favourable compared to the controls during the OP as it protects phenolics from early degradation. In the GP, the microcapsules showed a slightly increased TPC release compared to in the OP, while the release from the control samples decreased (Figure 5A–C). According to Figure 5A,C, the Cabernet Sauvignon and Merlot microcapsules showed comparable patterns, with the SA microcapsules releasing fewer TPCs than the control samples and the SA-GA and SA-GEL microcapsules releasing more TPCs than the control samples. As described by De Vos et al. [38] and Grgić et al. [8], the intended outcome of phenolics encapsulation is minimized release in the acidic gastric environment in order to preserve them for subsequent intestinal absorption. This is demonstrated by the sustained release of TPCs from the Cabernet Franc microcapsules during the GP (Figure 5B). In the IP, the control samples showed lower TPC release compared to in the GP, while the encapsulated samples demonstrated a gradual release of TPCs compared to the controls, with the Cabernet Franc SA microcapsules being the exception (Figure 5A–C). SA-GEL microcapsules consistently achieved the highest TPC release across all varieties, with values ranging from 9.30 to 28.92 mg_GAE_/100 mg_EXT_ (Figure 5A–C). Spray-drying significantly improved the stability and bioaccessibility of the TPCs, which was reflected in the *BI* values, which were higher than in the controls in most of the samples. A statistical analysis of the results of TPC release from the microcapsules during the simulation of in vitro digestion using repeated-measure ANOVA (*α* = 0.05) showed that there was no statistically significant difference in the dynamics of TPC release from microcapsules containing an extract of the same origin, regardless of the coating applied. On the other hand, it should be noted that encapsulation by spray-drying significantly improved the stability and bioaccessibility of TPCs, which was reflected in their *BI* values (Figure 5D). According to the Factorial ANOVA and Tukey HSD post hoc test (*p* < 0.05), the pomace variety and coating, as well as their interaction, had a statistically significant influence on the *BI* value for TPCs, with the coating having the greatest influence (54.4% of the variance), followed by pomace variety (30.4% of the variance) and the interaction between coating and variety (15.1%). The *BI* ranged from 18.9 to 23.9% for the control, 12.8 to 48.0% for the SA microcapsules, 25.2 to 82.4% for the SA-GA microcapsules and 37.8 to 110.8% for the SA-GEL microcapsules. The microcapsules containing Cabernet Franc pomace extract had a statistically significantly lower *BI* value compared to the CS and M microcapsules.

During the OP, the TFC for all microcapsules was lower than the respective controls (3.19–4.44 mg_CE_/100 mg_EXT_) across all varieties (Figure 6A–C). For example, the TFC release in the OP from SA microcapsules ranged from 0.29 to 1.44 mg_CE_/100 mg_EXT_, while SA-GA microcapsules had a TFC release of 0.20 to 0.67 mg_CE_/100 mg_EXT_ and SA-GEL microcapsules 0.72 to 0.99 mg_CE_/100 mg_EXT_. Since the early degradation of flavonoids is reduced and these compounds are preserved for the intestinal phase, which is more important for the absorption of nutrients, the lower release during the OP shown in the encapsulated samples is beneficial, as Thilakarathna and Rupasinghe [39] suggested. In addition, the controls showed an overall decrease in TFC release in the GP compared to the OP, also seen in the SA microcapsules of Cabernet Sauvignon and Merlot and the SA-GEL microcapsules of Cabernet Sauvignon and Cabernet Franc (Figure 6A–C). During the GP, the Cabernet Franc and Merlot microcapsules showed a lower TFC release than their control samples (Figure 6B,C). Since flavonoids are easily degraded in the acidic environment of the stomach, this low release is beneficial. By maintaining stability during the GP, the microcapsules ensure that the TFC is available for the intestinal phase, where absorption mainly occurs as reported by Hollman [40] and Kumar and Pandey [41]. Similar to the TPCs, repeated-measure ANOVA (*α* = 0.05) showed that there was no statistically significant difference in the dynamics of TFC release from microcapsules containing an extract of the same origin, regardless of the coating applied. In general, the bioaccessibility of the TFC was lower compared to the bioaccessibility of the TPCs. Thus, *BI* values of 3.2–5.3% were obtained for the control samples, and the SA microcapsules showed 2.1–5.9% BI, SA-GA showed 3.9–9.3% and SA-GEL microcapsules showed the highest *BI* values for the TFC between 5.8% and 12.8% (Figure 6D). The statistically significant effect of the coating on the *BI* of the TFC was also confirmed by Factorial ANOVA (post hoc Tukey HSD test, *p* < 0.05), where the effect of this factor on the variance was 55.5%. In this case, SA coating had no effect on the increase in the *BI* compared to the control sample, while SA-GA and especially SA-GEL had a statistically significant effect on the increase in *BI*. All three grape pomace varieties as well as the interactions between the factors (coating and variety) influenced the *BI* values for the TFC, with the effect of their influence being equal, i.e., 21.4% and 22.7% of the variance, respectively.

The release of TPA during the OP was consistently lower in the encapsulated samples compared to the control samples for all varieties, with the highest values obtained for the SA-coated microcapsules (0.12–0.53 mg/100 mg_EXT_) (Figure 7A–C). This reduction in TPA release during the OP is beneficial as it prevents the premature degradation of proanthocyanidins in the mouth where absorption does not occur [42,43]. TPA release in the GP was lower in all control samples than in the OP and remained minimal in all encapsulated samples with no discernible release trend (Figure 7A–C). This sustained low release is beneficial as it protects the proanthocyanidins from degradation in the acidic gastric environment and ensures their preservation for the intestinal phase where absorption predominantly occurs [43]. As with the GP, the release of TPA from the control samples decreased in the IP. Furthermore, no specific pattern was seen in the release of the TPA from the different microcapsules, which, as with the release of TPCs and the TFC during in vitro digestion, was confirmed by repeated-measure ANOVA (*α* = 0.05). It is evident that the bioaccessibility of TPA was the lowest compared to the other phenolic groups, i.e., its *BI* values ranged from 1.5 to 4.0% for the control samples, 1.0 to 2.8% for the SA microcapsules, 1.8 to 5.0% for SA-GA and 2.5 to 6.1% for SA-GEL (Figure 7D). Again, Factorial ANOVA (post hoc Tukey HSD test, *p* < 0.05) showed a statistically significant influence of both factors (coating and grape variety), with the interaction between the factors having the greatest influence on the *BI* (55.2% of the variance), followed by the coating (27.1% of the variance), while the variation in grape variety influenced the variance by 16.4%.

Overall, the SA-GEL microcapsules consistently showed the highest *BI* values for TPCs, TFC and TPA for all varieties, indicating that this combination of coatings provides better protection and higher release efficiency. Among the varieties, the encapsulated extracts of Cabernet Franc showed the lowest bioaccessibility for all three groups of phenols (TPCs, TFC, TPA).

#### 2.4.2. Bioaccessibility of Individual Phenolics

The *BI* values for the phenolic compounds observed at the end of the IP are shown in Table 3, while the Supplementary Materials (Tables S1–S3)  show a release profile of all quantifiable individual phenols in the microcapsules and the control samples at all stages of the in vitro digestion. The calculated *BI* values were analyzed using Factorial ANOVA, and statistically significant influences of the factor coating and the factor grape variety as well as their interactions on the *BI* of the individual phenolic compounds were found. The Tukey HSD post hoc test (*p* < 0.05) was used to determine the source of data variability. The results of the post hoc test for the “coating × variety” effect are shown in Table 3. The lower-case letters indicate a particular group of *BI* data for each phenolic compound.

*Phenolic acids:* The highest *BI* of gallic acid was determined for the samples of CS and M to be from the microcapsules SA-GA (117.0–222.6%) and SA-GEL (215.5–218.5%), whereby these values were significantly increased by 4.7–5.2-fold and 4.6–9.6-fold, respectively, compared to the controls. For 3,4-dihydroxybenzoic acid, the most significant increase in *BI* values of 2.1–19.3-fold was observed for all SA-GEL microcapsules compared to the controls. Syringic acid was mainly released in the oral and gastric phase, whereas all control and encapsulated CS samples showed no release in the intestinal phase, so a calculation of the *BI* was not possible in this case. However, syringic acid was detected in the IP for the CF and M microcapsules, with *BI* values of 59.1–102.3% and 36.8–537.8%, respectively. The *BI* values of vanillic acid improved with encapsulation, except for in the CF samples, with SA-GEL achieving the highest *BI* for CS (87.6%) and SA-GA for M (144.4%). Ellagic acid, which was minimally released from the control samples only in the IP, showed remarkable improvements in its *BI* values under SA-GEL and SA-GA microcapsules in sample M, with a highest *BI* of 107.9% and 90.6%, respectively. Spray-drying led to a significant improvement in the *BI* values of *o*-coumaric acid, especially from M-SA-GA and M-SA-GEL microcapsules, which reached 325.7% and 464.2%, respectively. However, *p*-coumaric acid, which was detected in all digestion phases in the control samples with *BI* values between 65.4 and 272.8%, was not present in the microcapsules, indicating possible limitations in *EE* or low initial concentrations. Finally, *p*-hydroxybenzoic acid, caffeic acid and ferulic acid were not released during the simulated digestion in all samples.

*Stilbenes and flavonoids:* No release was observed for the stilbenes, resveratrol and ε-viniferin tested in both the control and encapsulated samples (Tables S1–S3), indicating a significant susceptibility to digestive conditions, and consistent with the literature describing their sensitivity to pH and enzyme activity [44]. Although no release of the flavonol kaempferol was observed in any sample, two other flavonols studied, quercetin and rutin, were identified in the OP and GP in all control samples, but not in the encapsulated samples (Tables S1–S3).

*Flavanols:* Epicatechin was released differently depending on the grape variety and coating. Encapsulation improved its *BI*, especially for CS and M encapsulated with SA-GA and SA-GEL, which reached 53.5–97.5% and 83.6–139.2%, respectively. On the other hand, catechin was not detected in the intestinal phase, and its *BI* could not be calculated for any of the samples. Epicatechin gallate, which was not detected in the oral phase, showed a high *BI*, especially for CS (113.6–356.4%) and M microcapsules (367.8–476.4%). Gallocatechin gallate exhibited exceptionally high *BI*s after encapsulation, reaching 692.4–2028.7% for CS, 120.3–348.2% for CF and 580.7–1970.9% for M. Procyanidin B1, which was minimally released from the control samples in the OP and GP, showed improved *BI*s after encapsulation, reaching *BI* values of 71.2–155.1% for CS and 48.4–135.4% for M. Procyanidin B2 showed limited release in most samples, but its highest *BI* was obtained for M SA-GA microcapsules (283.8%). The lower stability of procyanidin B2 in the later stages of digestion could be due to its complexation with other dietary components or its rapid degradation [42,45].

*Anthocyanins:* Encapsulation improved the *BI* value of oenin chloride only in the CS–SA-GEL microcapsule (63.9%) compared to the control sample. Myrtillin chloride and petunidin chloride were only detected in the control samples in the OP and GP, and they were not detected in the IP. Kuromanin chloride was not detected in any of the samples. For peonidin-3-O-glucoside chloride, *BI* values were determined in all control samples (24.0–42.4%), and encapsulation did not improve these *BI* values in any of the microcapsules, indicating the low stability of this compound under the tested conditions. The results obtained are consistent with previous studies indicating the susceptibility of anthocyanins to digestive conditions, which could not be mitigated by encapsulation under the current formulation and process conditions [46].

In general, encapsulation had the greatest effect on increasing the *BI* of phenolic acids and flavanols (Table 3) compared to the control samples. The *BI* of 11 phenolic compounds was enhanced by adding an additional coating to SA, indicating possible interactions between the coating matrix and the phenolic compounds [47,48]. For example, the hydroxyl groups of gallic acid can form stabilizing hydrogen bonds with the gelatin in SA-GEL [49,50], while the emulsion-stabilizing properties of the gum Arabic in SA-GA create a protective environment for the syringic acid [51,52]. These compound–matrix interactions influence how effectively the individual phenolic acids are protected and released, and ultimately improve bioaccessibility, reflecting the ability of the coating combination to protect the active ingredients from early degradation and promote their release in the intestinal phase, where absorption is most favourable. According to the results, there were notable differences in the *BIs* of the phenolic compounds between grape varieties, with Merlot and Cabernet Sauvignon often showing greater *BI* improvements than Cabernet Franc. This suggests that the effectiveness of encapsulation is not uniform across varieties, likely due to differences in their chemical composition and the interactions of phenolic compounds with the coating matrices.

## 3. Conclusions

This study demonstrates how spray-drying phenol-rich grape pomace extracts (Cabernet Sauvignon, Cabernet Franc, and Merlot) using SA, SA-GA and SA-GEL coatings affects the release behaviour, bioaccessibility and physicochemical properties of their phenolic compounds. The findings emphasize the critical role of coating selection in achieving specific functional objectives.

SA-GEL and SA-GA coatings significantly improved *EE* (38.76–68.14% and 22.33–38.24%, respectively), with SA-GEL microcapsules being more uniform, being smaller in size and demonstrating the highest intestinal phenolic release (64.10–75.93%). Thermal and structural analyses confirmed the amorphous nature of all microcapsules.

Simulated digestion in vitro revealed that encapsulation improved the bioaccessibility of phenolic compounds, particularly phenolic acids and flavanols, with SA-GEL outperforming the other coatings due to the pH sensitivity of gelatin. However, anthocyanins and stilbenes exhibited lower stability, indicating a need for improved formulations. Grape variety also influenced bioaccessibility, with Merlot and Cabernet Sauvignon outperforming Cabernet Franc.

By valorizing grape pomace, this research promotes sustainable food processing while addressing the demand for functional ingredients. The limitations of this study include the need for the further optimization of formulations for industrial-scale production, the evaluation of long-term stability and a confirmation of bioactivity and physiological effects. Future research should explore these aspects and investigate the integration of these formulations into commercial products to support the development of sustainable, health-promoting food products.

## 4. Materials and Methods

### 4.1. Materials

The chemicals for encapsulation (coatings SA, GA, GEL) and in vitro digestion (pepsin, pancreatin enzymes and porcine bile extract) were purchased from Sigma Aldrich (Saint Louis, MO, USA). Reagents for the spectrophotometric determination of TPCs, TFC and TPA were purchased from CPA chem (Bogomilovo, Bulgaria), Alfa Aesar GmbH & Co KG (Kandel, Germany) and Acros Organics (Geel, Belgium). Authentic standards of phenolic acids, flavonols, flavan-3-ols, stilbenes and anthocyanins were purchased from Sigma Aldrich (Saint Louis, MO, USA), Extrasynthese (Genay, France), Acros Organics (Geel, Belgium) and Applihem (Darmstadt, Germany) [23].

Grape pomace left over after the processing of Cabernet Sauvignon, Cabernet Franc and Merlot grapes into wine at the Erdut winery (Erdutski vinogradi d.o.o., Erdut, Croatia) was used. The collected grape pomace, consisting of seeds, skin, and pulp, was air-dried at room temperature for 48 h and stored in closed containers at room temperature before use. Prior to the experiments, the dried pomace was milled to a particle size of ≤1 mm using an ultracentrifugal mill (Retsch ZM200, Haan, Germany). The dry matter content of the pomace was measured and recorded as follows: Cabernet Sauvignon—92.91 ± 0.01%; Cabernet Franc—91.95 ± 0.02%; and Merlot—92.15 ± 0.04%.

### 4.2. Extraction of Phenolic Compounds from Grape Pomace

Conventional extraction of the phenolic compounds from the pomace samples (1 g) was carried out with 40 mL of a 50% ethanol solution at 80 °C with continuous stirring at 200 rpm in a shaking water bath (Julabo, SW-23, Seelbach, Germany) for 120 min. After extraction, the mixture was centrifuged at 11,000× *g* for 10 min (Z 326 K, Hermle Labortechnik GmbH, Wehingen, Germany) to separate the supernatant [53].

The supernatant was concentrated under vacuum (48 mbar/50 °C; Büchi, R-210, Flawil, Switzerland) by halving the volume to minimize the ethanol content. The removed ethanol was replaced by an appropriate amount of redistilled water, resulting in a phenol-rich pomace extract, which was subjected to spray-dried encapsulation.

### 4.3. Spray-Drying Microencapsulation

The encapsulation mixture, consisting of the extract and the coating(s), was prepared in a glass vessel using a magnetic stirrer (Witeg Labortechnik GmbH, Wertheim, Germany) at room temperature for 24 h. The concentration of SA was 1% (*w*/*v*), while the concentration of GA was 0.53% (*w*/*v*) and GEL 1.67% (*w*/*v*). The spray-drying of the prepared mixture was performed using a B-290 mini spray-dryer (BÜCHI Labortechnik AG, Flawil, Switzerland) equipped with a 1.5 mm nozzle cap and a 0.5 mm needle. The process was carried out at an inlet air temperature of 180 °C and an outlet temperature of 89 °C. The pumping rate was set to 25%, with an approximate air flow of 600 L/h. The resulting microcapsules (MCs) were collected in a separating flask and then stored in sealed sample containers at 4 °C in the dark until further analysis.

### 4.4. Characterization of Microcapsules

#### 4.4.1. Encapsulation Efficiency Determination

The *EE* (%) was determined by measuring the total phenolic content in the microcapsules (TPC_MC_, mg_GAE_/g_db_) and the surface phenolic content (SPC_MC_, mg_GAE_/g_db_) based on the method described by Vu et al. [54] using the following Equation (1):

(1) $$EE\left(\%\right)=\frac{{TPC}_{MC}-{SPC}_{MC}}{{TPC}_{MC}}\times 100$$

As described by Tolun et al. [55], the samples were prepared for the determination of TPC_MC_ and SPC_MC_. Briefly, for TPC_MC_, 15 mg of MC was vortexed with 3 mL of an ethanol/glacial acetic acid/water solution (50:8:42, *v*/*v*/*v*), and the mixture was filtered through a 0.45 μm PTFE filter. For SPC_MC_, 24 mg of MC was dissolved in 3 mL ethanol/methanol (1:1, *v*/*v*) and also filtered through a 0.45 μm PTFE filter.

After the filtration of the samples, TPC_MC_ and SPC_MC_ were determined using the Folin–Ciocalteu method as described in Section 4.5.1. The results were expressed as gallic acid equivalents per MC dry basis (mg_GAE_/g_db_) ± SD.

#### 4.4.2. Scanning Electron Microscopy

Scanning electron microscopy (SEM) with a Hitachi S4700 instrument (Hitachi Scientific Ltd., Tokyo, Japan) at 10 kV was used to analyze the morphology of the microcapsules. Before imaging, a thin layer of gold–palladium was sputtered onto the samples.

#### 4.4.3. Particle Size Analysis

Particle size analysis was conducted using Fiji (ImageJ) software (ImageJ 1.54 g, Java 11.8.0_322). The SEM images were calibrated based on the provided scale bar of 100 µm. The projected 2D area of each particle was measured, and the equivalent circular diameter was calculated using Equation (2):

(2) $$Diameter\left(µm\right)=2\times \sqrt{\frac{Area}{\pi }}$$

where *Area* is the 2D projected area of the particle in µm^2^.

The particle size distributions were expressed in terms of D10, D50 (median), D90 and the mean diameter ± standard deviation. The size range for each sample was also recorded to evaluate uniformity.

#### 4.4.4. X-Ray Powder Diffraction

The structural properties of the coatings and microcapsules were analyzed using an X-ray powder diffractometer (BRUKER D8 Advance diffractometer, Karlsruhe, Germany) with Cu Kα radiation at λ = 1.5406 Å over an angular range of 3–40° 2θ. Scanning was performed at 40 kV and 40 mA, and the VANTEC-1 detector was used to generate diffractograms. Data processing, including background removal, smoothing and Kα2 stripping, was performed using DIFFRAC plus EVA software (version 13.0.0.1, Karlsruhe, Germany).

#### 4.4.5. Differential Scanning Calorimetry

The thermal behaviour of the coatings and microcapsules was analyzed using a differential scanning calorimeter (Mettler Toledo 821e DSC; Mettler Inc., Schwerzenbach, Switzerland). The samples (3–5 mg), placed in aluminium pans, were heated at a constant rate of 10 °C/min in the temperature range of 25–300 °C under a constant argon flow of 150 mL/min. The thermograms were analyzed using STAR^e^ software (version 9.3, Mettler Toledo; Mettler Inc., Schwerzenbach, Switzerland).

### 4.5. Determination of Phenolic Compounds

#### 4.5.1. Total Phenolic Content

The TPCs were analyzed according to the modified method proposed by Waterhouse [56]. A mixture of 40 µL of sample, 3160 µL distilled water, 200 µL Folin–Ciocalteu reagent and 600 µL sodium carbonate (20% *w*/*v*) was incubated at 40 °C for 30 min. The absorbance was read at 765 nm against a solvent blank and the results were expressed as the mean of three replicates ± standard deviation (SD) as gallic acid equivalents per weight of extracts (mg_GAE_/100 mg_EXT_).

#### 4.5.2. Total Flavonoid Content

TFC was determined using a modified aluminium chloride method [57]. A sample of 500 µL was mixed with 2 mL water, 150 µL 5% sodium nitrite and 150 µL 10% aluminium chloride, and after 6 min with 1 mL 1 M sodium hydroxide. The mixture was diluted with distilled water (1.2 mL) to a total volume of 5 mL and shaken, and the absorbance was read at 510 nm. The results were expressed as the mean of three replicates ± standard deviation (SD) as (+)-catechin equivalents per weight of extracts (mg_CE_/100 mg_EXT_).

#### 4.5.3. Total Extractable Proanthocyanidin Content

TPA was measured using a modified acid–butanol reaction method [58]. A mixture of ferrous sulphate heptahydrate solution and HCl-butanol (2:3, *v*/*v*) was added to 500 µL of the sample. After incubation at 95 °C for 15 min and cooling, the absorbance was read at 540 nm. TPA was calculated using the molar weight of cyanidin and the extinction coefficient and was expressed in mg per 100 mg of extracts as the mean of three replicates ± standard deviation (SD).

#### 4.5.4. Individual Phenolic Compound Determination

The content of individual phenolic compounds (phenolic acids, flavanols, flavonols, stilbenes and anthocyanins) in the control samples and the prepared microcapsules was analyzed using ultra-high-performance liquid chromatography (UHPLC Nexera XR, Shimadzu, Japan) with a photodiode detector. A reversed-phase Kinetex^®^ C18 core–shell column (100 × 4.6 mm, 2.6 µm, Phenomenex, Torrance, CA, USA) was used for the separation. Samples were dissolved in appropriate solvents and concentrations, then filtered through 0.45 µm membranes and analyzed using LabSolutions software (version 5.87). Individual phenolics were identified and quantified by comparing their UV–Vis spectra and retention times with authentic standards under the same chromatographic conditions. Calibration curves were generated using external standards. The results, expressed as average of triplicates ± standard deviation (SD), were obtained from all measurements.

Phenolic acids, flavanols, flavonols and stilbenes were quantified according to the method of Bucić-Kojić et al. [59]. A linear gradient of two mobile phases was used: Phase A (1.0% acetic acid in water) and Phase B (methanol–acetonitrile, 50:50, *v*/*v*). Chromatography was performed at 30 °C with a flow rate of 1 mL/min, with the injection volume of the sample set to 20 µL.

Anthocyanins were analyzed according to Bucić-Kojić et al. [60] using mobile phases A (water/formic acid/acetonitrile, 87:10:3, *v*/*v*/*v*) and B (water/formic acid/acetonitrile, 40:10:50, *v*/*v*/*v*). The injection volume was 20 µL at a flow rate of 0.8 mL/min.

### 4.6. Phenolic Compound Release and Bioaccessibility Studies

#### 4.6.1. Enzyme-Free Release Study

This study followed the modified INFOGEST protocol [61] described by Martinović et al. [22] and observed the release of phenolic compounds over 243 min. The process was carried out at 37 °C with constant stirring.

OP: A total of 200 mg of the microcapsules, 4 mL of simulated salivary fluid (SSF) and 25 µL of CaCl_2_(H_2_O)_2_ were mixed. The pH was adjusted to 7 and the volume was made up to 10 mL with redistilled water. After 3 min, 2 mL was withdrawn for TPC analysis and replaced with SSF.

GP: A total of 8 mL of simulated gastric fluid (SGF) and 5 µL of CaCl_2_(H_2_O)_2_ were added and the pH was lowered to 3 with 1 M HCl. The final volume was adjusted to 20 mL. Over a period of 120 min, samples were taken at specific intervals (6th, 8th, 13th, 23rd, 48th, 63rd and 123rd minute) and the SGF was replenished.

IP: A total of 16 mL of simulated intestinal fluid (SIF) and 40 µL of CaCl_2_(H_2_O)_2_ were added and the pH was adjusted to 7. The volume was increased to 40 mL and this phase lasted 120 min, with samples being taken at specific intervals (126th, 128th, 133rd, 143rd, 168th, 183rd and 243rd minute) and the SIF replenished as required.

The First order, Higuchi, Hixson–Crowell and Korsmeyer–Peppas mathematical models were applied to analyze the release of phenols during the enzyme-free in vitro study. Data analysis was performed using DDSolver [62], and the best-fitting model was determined using the adjusted R^2^ (R^2^_adj_), the Akaike information criterion (AIC) and the model selection criterion (MSC).

#### 4.6.2. In Vitro Simulated Digestion and Determination of Bioaccessibility Index

In vitro digestion with enzymes followed the modified INFOGEST protocol, as described by Martinović et al. [22]. Test tubes representing specific digestion time points (OP_3_, GP_63_, GP_123_, IP_163_, IP_243_) were mixed with a vertical rotator (PTR-60, Grant-bio Instruments, Royston, UK) in a thermostat (TC 135 S, Lovibond, Dortmund, Germany) set to 37 °C.

For the OP, 100 mg of the control sample or 200 mg of the microcapsules was mixed with 4 mL of simulated saliva fluid (SSF) and 25 µL of CaCl_2_(H_2_O)_2_. The pH was adjusted to 7 and the total volume was made up to 10 mL with distilled water. After 3 min, the OP test tube (OP_3_) was removed and the gastric phase (GP) began in the remaining tubes by adding 8 mL of simulated gastric fluid (SGF), 5 µL of CaCl_2_(H_2_O)_2_, 500 µL of pepsin and water to a total volume of 20 mL. The samples were taken at specific time points (GP_63_, GP_123_). The IP began with the addition of 8.5 mL simulated intestinal fluid (SIF), 40 µL CaCl_2_(H_2_O)_2_, 5 mL pancreatin solution, 2.5 mL bile extract solution and water to adjust the volume to 40 mL [23]. Samples were collected at IP_163_ and IP_243_ to complete the digestion process. After removal, the test tubes were centrifuged (16,000× *g*, 4 °C, 30 min) and the supernatant was filtered through 0.45 μm membranes (Syringe filters Spheros Nylon, Agilent Technologies, Santa Clara, CA, USA).

To remove impurities such as salts, bile and enzyme residues, solid phase extraction was performed according to a modified method of the one by Kamiloglu et al. [63], as described by Martinović et al. [22]. The prepared samples were then analyzed for TPCs, TFC, TPA and individual phenolic compounds.

The bioaccessibility index (*BI*, %) was calculated using the following Equation (3):

(3) $$BI\;\left(\%\right)=\frac{C_{A}}{C_{B}}\times 100$$

where *C*_A_ is the content of phenolic compounds after complete digestion (IP_243_) and *C*_B_ is the content before digestion in the control sample, expressed per 100 mg extract.

### 4.7. Statistical Analysis

Factorial ANOVA (two-way ANOVA) was performed using TIBCO Statistica software version 14.0.0.15 (TIBCO Software Inc., Palo Alto, CA, USA) to assess the statistical significance of differences between sample means for *EE* and *BI* for TPC, TFC, TPA and individual phenols. When significant differences were found, the Tukey HSD test was applied as a post hoc analysis to identify specific sample groups with significant differences (*p* < 0.05). Repeated-measure ANOVA was used to evaluate the effects of encapsulation with different coatings on the release of TPCs, TFC and TPA during simulated in vitro digestion.

## Figures and Tables

**Table 1:** Mean diameter (±standard deviation) and particle size distribution (D10, D50, D90) of spray-dried microcapsules prepared with different grape pomace extracts and coatings (SA—sodium alginate; SA-GA—combination of sodium alginate with gum Arabic; SA-GEL—combination of sodium alginate with gelatin).

Grape Variety	Coatings	Mean Diameter (µm)	D10 (µm)	D50 (µm)	D90 (µm)	Range (µm)
Cabernet Sauvignon	SA	3.96 ± 2.20	2.08	3.03	6.95	1.97–11.98
	SA-GA	2.61 ± 1.73	1.25	1.95	5.36	1.19–10.69
	SA-GEL	2.62 ± 1.91	1.24	1.82	5.35	1.18–12.02
Cabernet Franc	SA	4.29 ± 2.26	2.10	3.46	7.37	1.97–11.90
	SA-GA	2.59 ± 1.71	1.24	1.82	5.51	1.18–10.08
	SA-GEL	2.58 ± 1.81	1.24	1.87	5.21	1.18–12.17
Merlot	SA	2.82 ± 2.12	1.24	1.95	5.81	1.19–12.23
	SA-GA	2.53 ± 1.69	1.24	1.86	4.94	1.18–11.56
	SA-GEL	2.38 ± 1.72	1.18	1.67	4.57	1.18–12.31

**Table 2:** Estimated parameters of the mathematical models used to describe the release kinetics of the total phenolic compounds from microcapsules containing grape pomace extracts prepared with various coatings, as well as statistical criteria for model approximation success.

Mathematical Models		Release Rate Constants and Statistical Criteria of Model Approximation Success								
		Cabernet Sauvignon			Cabernet Franc			Merlot		
		SA	SA-GA	SA-GEL	SA	SA-GA	SA-GEL	SA	SA-GA	SA-GEL
First order model	R^2^_adj_	0.855	0.779	0.784	0.937	0.916	0.759	0.929	0.899	0.765
	AIC	123.918	130.018	128.235	113.001	117.112	131.785	114.313	119.575	133.050
	MSC	1.421	0.813	1.225	2.272	1.921	1.115	2.140	1.846	1.198
	*k*_1_	0.031	0.041	0.008	0.042	0.042	0.010	0.036	0.029	0.009
Higuchi model	R^2^_adj_	0.707	0.418	0.852	0.788	0.693	0.833	0.806	0.887	0.801
	AIC	135.664	145.806	122.179	132.628	138.007	125.930	130.329	121.545	130.400
	MSC	0.687	−0.174	1.603	1.046	0.615	1.481	1.139	1.723	1.363
	*k*_H_	8.034	8.588	5.537	8.354	8.543	5.849	8.081	7.776	5.438
Hixson–Crowell model	R^2^_adj_	0.006	0.362	0.721	0.744	0.639	0.727	0.772	0.816	0.721
	AIC	0.679	147.195	132.287	135.667	140.634	133.734	132.901	129.430	135.799
	MSC	137.111	−0.261	0.971	0.856	0.451	0.993	0.979	1.230	1.026
	*k*_HC_	0.596	0.007	0.003	0.007	0.007	0.003	0.007	0.006	0.003
Korsmeyer– Peppas model	R^2^_adj_	0.913	0.886	0.741	0.872	0.873	0.688	0.939	0.974	0.652
	AIC	117.081	119.646	132.021	122.718	122.287	136.782	112.575	98.676	140.272
	MSC	1.848	1.461	0.988	1.665	1.598	0.803	2.249	3.152	0.746
	*k*_KP_	20.189	35.888	10.596	18.357	26.022	12.519	21.047	17.533	7.605
	*n*	0.319	0.204	0.341	0.356	0.272	0.313	0.316	0.335	0.391

(*k*_1_, *k*_H_, *k*_HC_, *k*_KP_—release constants for the corresponding model; *n*—diffusion exponent; R^2^_adj_—adjusted coefficient of determination; AIC—Akaike information criterion; MSC—model selection criterion) [23].

**Table 3:** *BI* (%) of the individual phenolic compounds of the control samples (C) and the microencapsulated extracts from different grape pomaces (Cabernet Sauvignon (CS), Cabernet Franc (CF), Merlot (M)).

Phenolics		Sample			*BI* (%) *			
			CS		CF		M	
Phenolic acids	Gallic acid	C	22.4 ± 0.8	c	108.8 ± 4.9	b	47.4 ± 0.3	c
		SA	42.8 ± 3.7	c	36.9 ± 0.4	c	26.9 ± 0.7	c
		SA-GA	117.0 ± 4.4	b	18.2 ± 0.0	c	222.6 ± 7.3	a
		SA-GEL	215.5 ± 4.4	a	95.7 ± 0.4	a	218.5 ± 27.7	a
	3,4-Dihydroxybenzoic acid	C	18.3 ± 0.6	e	70.6 ± 0.4	cd	12.3 ± 0.2	e
		SA	61.7 ± 2.9	d	67.1 ± 3.7	cd	66.9 ± 1.2	cd
		SA-GA	77.0 ± 7.5	c	74.3 ± 2.8	cd	157.8 ± 2.7	b
		SA-GEL	226.6 ± 3.8	a	149.0 ± 3.8	b	236.8 ± 5.7	a
	Syringic acid	C	0.0	d	0.0	d	0.0	d
		SA	0.0	d	2.7 ± 0.0	cd	6.8 ± 0.2	c
		SA-GA	0.0	d	1.6 ± 0.1	cd	94.0 ± 1.5	a
		SA-GEL	0.0	d	0.0	d	28.0 ± 5.5	b
	Vanillic acid	C	22.9 ± 1.1	f	29.6 ± 0.0	f	22.1 ± 0.7	f
		SA	53.0 ± 0.7	de	9.5 ± 0.8	g	49.3 ± 0.0	e
		SA-GA	58.5 ± 2.0	d	22.6 ± 0.5	f	144.4 ± 4.1	a
		SA-GEL	87.6 ± 2.8	b	24.0 ± 0.0	f	72.4 ± 4.6	c
	Ellagic acid	C	1.7 ± 0.1	d	1.6 ± 0.0	d	15.5 ± 0.5	cd
		SA	9.0 ± 0.4	d	0.0	d	70.2 ± 2.3	b
		SA-GA	15.0 ± 1.8	cd	7.9 ± 0.2	d	90.6 ± 7.7	ab
		SA-GEL	38.1 ± 0.2	c	8.4 ± 0.4	d	107.9 ± 18.5	a
	*o*-Coumaric acid	C	78.7 ± 12.4	d	48.1 ± 0.3	d	61.7 ± 0.6	d
		SA	191.0 ± 15.8	c	63.8 ± 3.0	d	57.8 ± 14.3	d
		SA-GA	287.5 ± 37.2	bc	19.5 ± 1.7	d	325.7 ± 6.6	b
		SA-GEL	337.2 ± 31.5	b	54.8 ± 1.8	d	464.2 ± 66.8	a
	*p*-Coumaric acid	C	87.0 ± 3.3	b	272.8 ± 49.0	a	65.4 ± 2.5	b
		SA	0.0	c	0.0	c	0.0	c
		SA-GA	0.0	c	0.0	c	0.0	c
		SA-GEL	0.0	c	0.0	c	0.0	c
Flavanols	Epicatechin	C	49.8 ± 1.1	c	0.0	d	15.6 ± 0.2	d
		SA	57.2 ± 1.2	c	3.6 ± 0.1	d	0.0	d
		SA-GA	97.5 ± 13.0	b	4.9 ± 0.8	d	53.5 ± 0.7	c
		SA-GEL	139.2 ± 1.8	a	8.8 ± 0.4	d	83.6 ± 4.7	b
	Epicatechin gallate	C	116.8 ± 13.2	cd	98.8 ± 0.2	cde	88.7 ± 8.8	cde
		SA	113.6 ± 24.0	cde	5.8 ± 0.3	e	367.8 ± 45.1	ab
		SA-GA	186.3 ± 28.5	c	36.7 ± 3.7	de	461.0 ± 25.8	ab
		SA-GEL	356.4 ± 10.2	b	19.6 ± 1.6	de	476.4 ± 68.3	a
	Gallocatechin gallate	C	251.4 ± 10.4	h	150.0 ± 4.7	j	234.0 ± 0.2	hi
		SA	692.4 ± 22.1	e	120.3 ± 0.5	j	580.7 ± 17.3	f
		SA-GA	980.7 ± 17.9	d	197.6 ± 8.7	i	1342.7 ± 10.3	c
		SA-GEL	2028.7 ± 15.0	a	348.2 ± 0.9	g	1970.9 ± 1.7	b
	Procyanidin B1	C	0.0	d	0.0	d	0.0	d
		SA	71.2 ± 8.1	c	0.0	d	48.4 ± 4.5	c
		SA-GA	130.4 ± 4.1	b	0.0	d	119.1 ± 7.1	b
		SA-GEL	155.1 ± 6.2	a	0.0	d	135.4 ± 14.9	ab
	Procyanidin B2	C	0.0	b	0.00	b	0.0	b
		SA	0.0	b	11.1 ± 1.5	b	0.0	b
		SA-GA	0.0	b	9.7 ± 0.1	b	283.8 ± 10.5	a
		SA-GEL	0.0	b	0.0	b	0.0	b
Flavonols	Rutin	C	0.0	b	29.0 ± 2.8	a	0.0	b
		SA	0.0	b	0.0	b	0.0	b
		SA-GA	0.0	b	0.0	b	0.0	b
		SA-GEL	0.0	b	0.0	b	0.0	b
Anthocyanins	Oenin chloride	C	34.4 ± 2.8	bc	32.7 ± 2.9	bc	62.6 ± 4.5	a
		SA	9.1 ± 1.4	e	4.7 ± 0.1	e	14.9 ± 0.2	de
		SA-GA	28.0 ± 3.6	cd	3.4 ± 0.3	e	40.1 ± 0.0	bc
		SA-GEL	63.9 ± 10.5	a	10.2 ± 0.7	e	44.7 ± 4.6	b
	Peonidin-3-*O*-glucoside chloride	C	24.7 ± 4.3	b	24.0 ± 2.9	b	42.4 ± 5.9	a
		SA	0.0	c	2.5 ± 0.3	c	0.0	c
		SA-GA	0.0	c	0.0	c	0.0	c
		SA-GEL	0.0	c	0.0	c	0.0	c

* *BI* (%) is given as mean ± standard deviation (SD). Different lower-case letters indicate statistically significant differences between groups for each phenol according to Factorial ANOVA and Tukey HSD post hoc test (*p* < 0.05).

## Data Availability

The original contributions presented in this study are included in the article/Supplementary Material. Further inquiries can be directed to the corresponding author.

## Supplementary Materials

The following supporting information can be downloaded at https://www.mdpi.com/article/10.3390/gels11020130/s1. Figure S1: TPC release from differently coated extracts of different grape pomaces (A.—Cabernet Sauvignon; B.—Cabernet Franc; and C.—Merlot) during simulated digestion without enzymes, expressed as gallic acid equivalent per microcapsule mass; Figure S2: Kinetics of TPC release from microencapsulated extracts with different coatings: SA (A.); SA-GA (B.); SA-GEL (C.) (symbols—experimental data; lines—approximate curves according to different mathematical models); Table S1. Release profile of individual phenolics from Cabernet Sauvignon control extract (C_CS_) and from SA, SA-GA and SA-GEL microcapsules before digestion (BD) and during all phases of simulated in vitro digestion; Table S2. Release profile of individual phenolics from Cabernet Franc control extract (C_CF_) and from SA, SA-GA and SA-GEL microcapsules before digestion (BD) and during all phases of simulated in vitro digestion; Table S3. Release profile of individual phenolics from Merlot control extract (CM) and from SA, SA-GA and SA-GEL microcapsules before digestion (BD) and during all phases of simulated in vitro digestion.
