# Supplementary material for: Spray-Drying Microencapsulation of Grape Pomace Extracts with Alginate-Based Coatings and Bioaccessibility of Phenolic Compounds
Source: Gels. 2025 Feb 11;11(2):130. doi: 10.3390/gels11020130 (PMC11854297; doi:10.3390/gels11020130)
Supplement: Supplementary file 1 [file gels-11-00130-s001.zip › gels-3441115-supplementary.pdf]

gels

MDPI

Article

Spray-Drying Microencapsulation of Grape Pomace Extracts with Alginate-Based Coatings by and Bioaccessibility of Phenolic Compounds

Josipa Martinović [1], Rita Ambrus [2], Mirela Planinić [1], Gabriela Perković [1], Gordana Šelo [1], Ana-Marija Klarić [1] and Ana Bucić-Kojić [1,*]

[1] Faculty of Food Technology Osijek, Josip Juraj Strossmayer University of Osijek, F. Kuhača 18, HR-31000 Osijek, Croatia; mplanini@ptfos.hr (M.P.); gperkovic@ptfos.hr (G.P.); gselo@ptfos.hr (G.Š.); ana-marija.klaric@ptfos.hr (A.-M.K.)

[2] Faculty of Pharmacy, Institute of Pharmaceutical Technology and Regulatory Affairs, University of Szeged, H-6720 Szeged, Hungary; ambrus.rita@szte.hu

* Correspondence: abucic@ptfos.hr; Tel.: +385-31-224-334

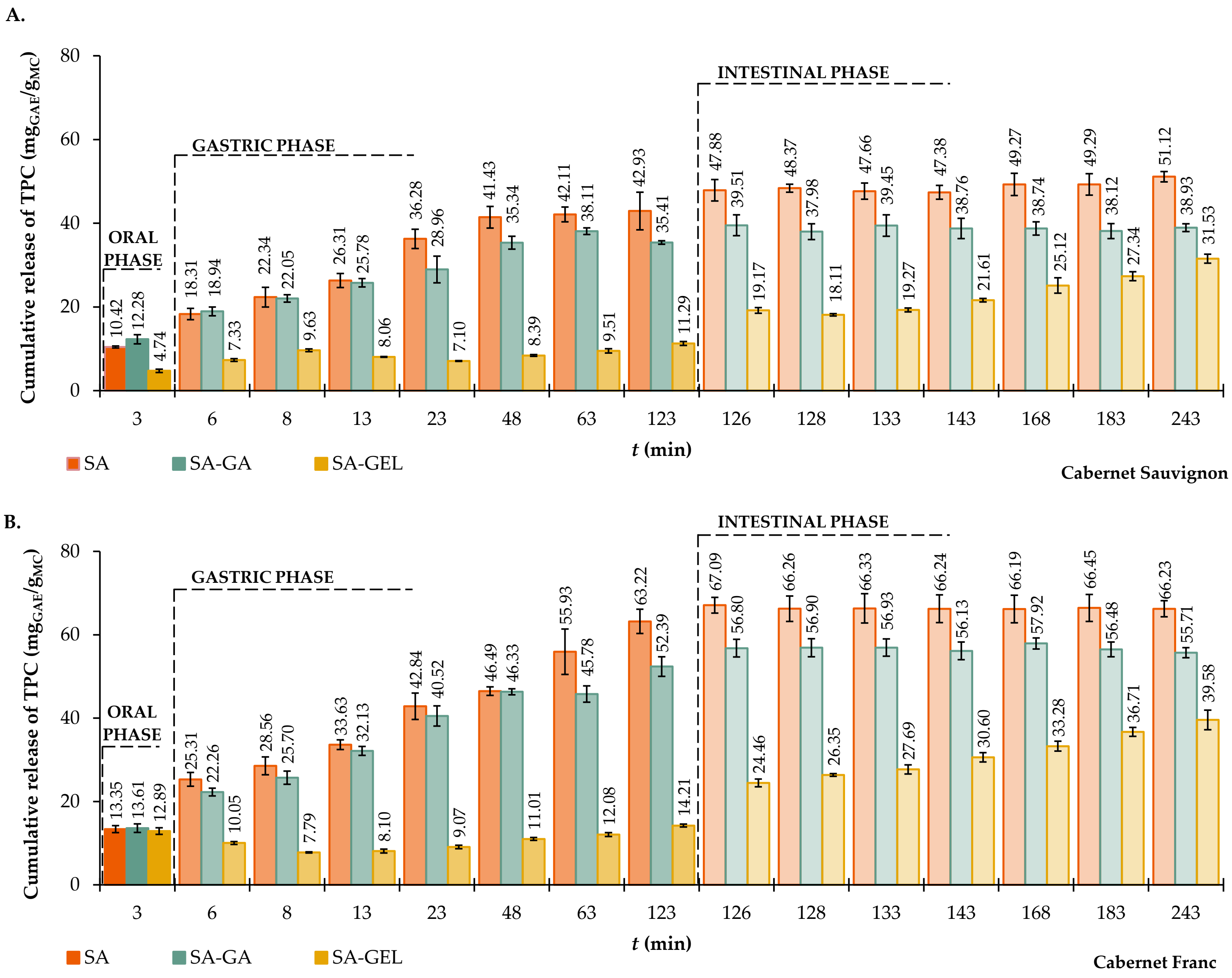
A.
Cumulative release of TPC (mgGAE/gMC)
0
20
40
60
80
ORAL PHASE
GASTRIC PHASE
INTESTINAL PHASE
10.42
12.28
4.74
18.31
18.94
7.33
22.34
22.05
9.63
26.31
25.78
8.06
36.28
28.96
7.10
41.43
35.34
8.39
42.11
38.11
9.51
42.93
35.41
11.29
47.88
39.51
19.17
48.37
37.98
18.11
47.66
39.45
19.27
47.38
38.76
21.61
49.27
38.74
25.12
49.29
38.12
27.34
51.12
38.93
31.53
3
6
8
13
23
48
63
123
126
128
133
143
168
183
243
t (min)
SA
SA-GA
SA-GEL
Cabernet Sauvignon
B.
Cumulative release of TPC (mgGAE/gMC)
0
20
40
60
80
ORAL PHASE
GASTRIC PHASE
INTESTINAL PHASE
13.35
13.61
12.89
25.31
22.26
10.05
28.56
25.70
7.79
33.63
32.13
8.10
42.84
40.52
9.07
46.49
46.33
11.01
55.93
45.78
12.08
63.22
52.39
14.21
67.09
56.80
24.46
66.26
56.90
26.35
66.33
56.93
27.69
66.24
56.13
30.60
66.19
57.92
33.28
66.45
56.48
36.71
66.23
55.71
39.58
3
6
8
13
23
48
63
123
126
128
133
143
168
183
243
t (min)
SA
SA-GA
SA-GEL
Cabernet Franc

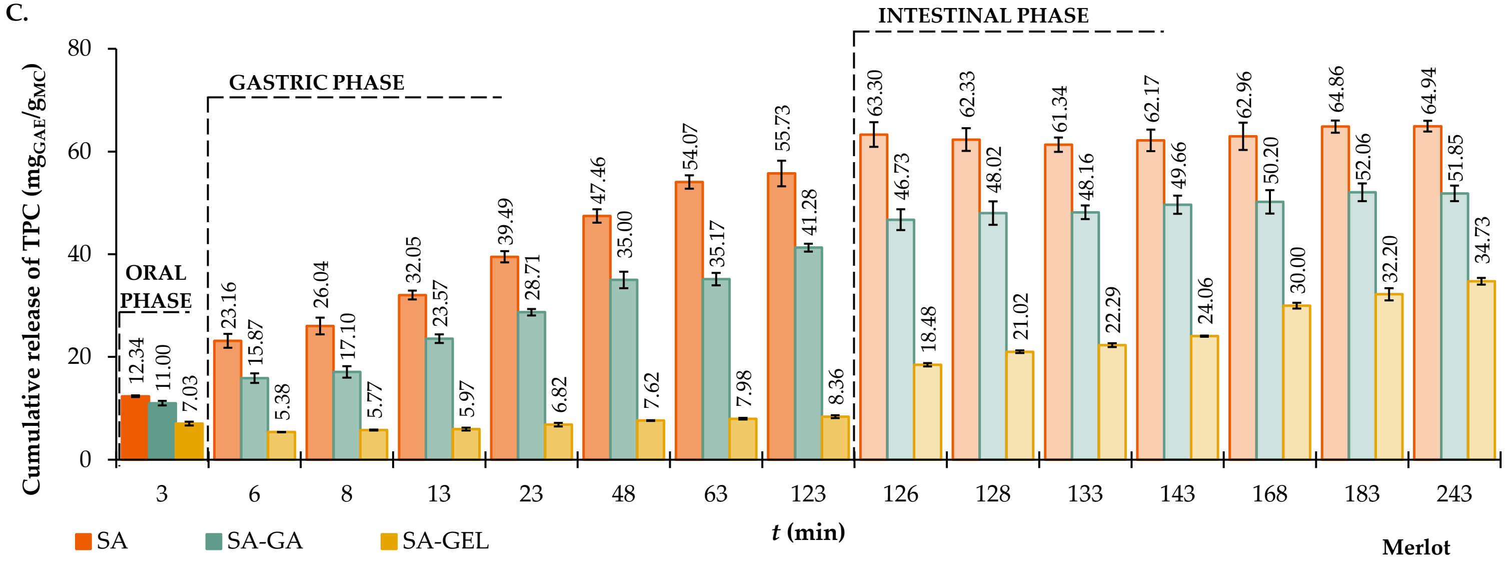

Figure S1. TPC release from differently coated extracts of different grape pomace (A. – Cabernet Sauvignon, B. – Cabernet Franc and C. – Merlot) during simulated digestion without enzymes, expressed as gallic acid equivalent per microcapsule mass.

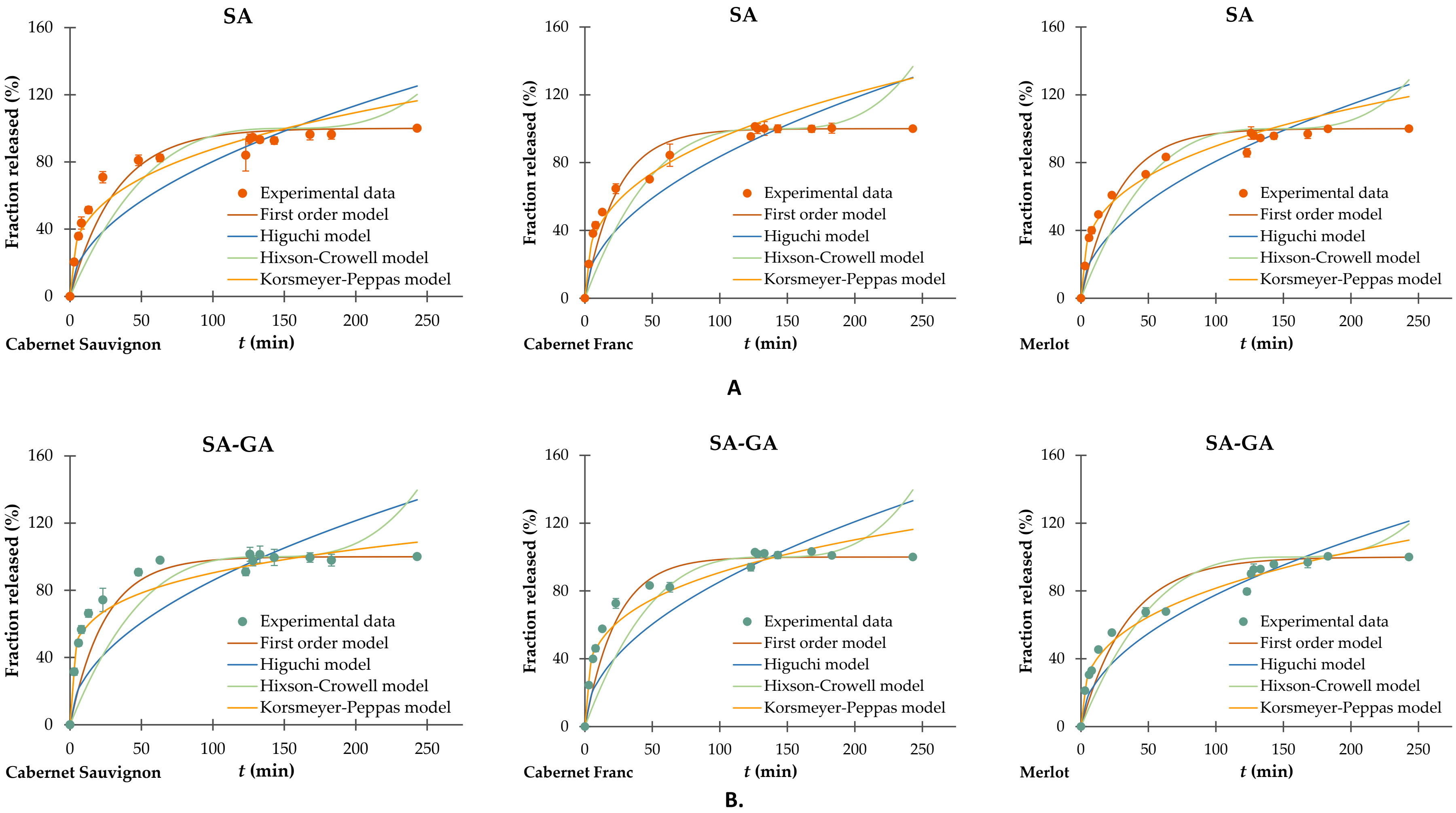
SA
Fraction released (%)
160
120
80
40
0
0
50
100
150
200
250
Cabernet Sauvignon
t (min)
Experimental data
First order model
Higuchi model
Hixson-Crowell model
Korsmeyer-Peppas model
SA
Cabernet Franc
SA
Merlot
A
SA-GA
Cabernet Sauvignon
SA-GA
Cabernet Franc
SA-GA
Merlot
B.

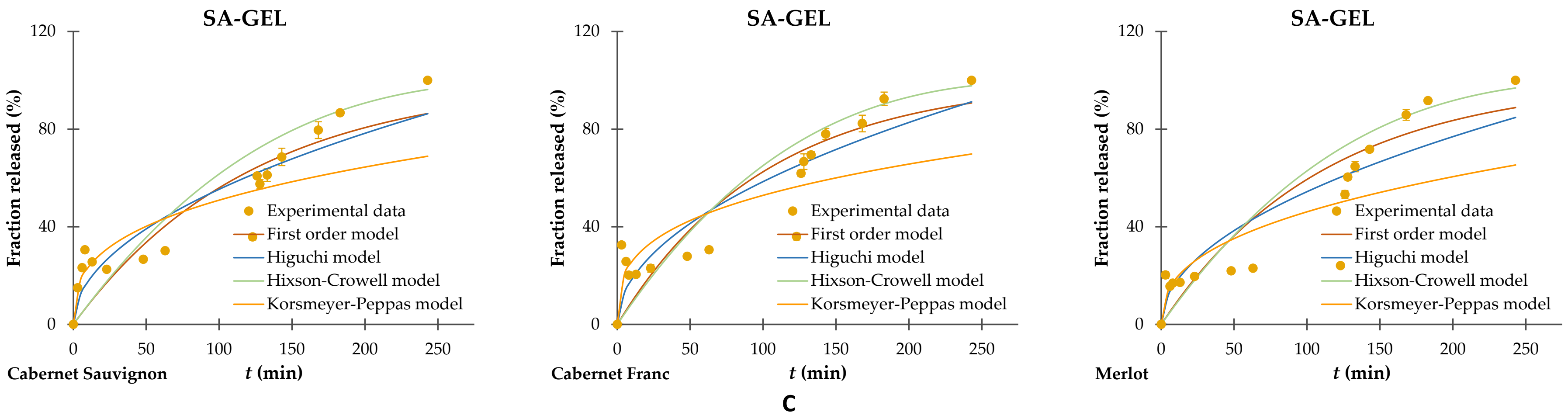

Figure S2. Kinetics of TPC release from the microencapsulated extracts with different coatings SA (**A.**); SA-GA (**B.**); SA-GEL (**C.**) (symbols – experimental data, lines – approximate curves according to different mathematical models).

gels

MDPI

Table S1. Release profile of individual phenolics from Cabernet Sauvignon control extract (C_{CS}) and from SA, SA-GA and SA-GEL microcapsules before digestion (BD) and during all phases of simulated in vitro digestion.

**Component**	**Sample**	**Before digestion**	**Oral phase**	**Gastric phase**		**Intestinal phase**	
		**BD**	**OP_3**	**GP_{63}**	**GP_{123}**	**IP_{183}**	**IP_{243}**
***Phenolic acids* (µg/100 mg_{EXT})**							
Gallic acid	C_{CS}	161.99 ± 31.68	0.70 ± 0.04	-	1.25 ± 0.01	-	36.26 ± 1.29
	SA		nd	nd	nd	103.19 ± 1.10	69.28 ± 6.02
	SA-GA		nd	nd	nd	144.67 ± 10.70	189.45 ± 7.20
	SA-GEL		nd	nd	nd	264.25 ± 6.74	349.09 ± 7.06
3,4-Dihydroxybenzoic acid	C_{CS}	62.02 ± 11.60	nd	-	nd	-	11.36 ± 0.37
	SA		nd	nd	6.71 ± 1.04	42.42 ± 0.35	38.29 ± 1.80
	SA-GA		nd	nd	nd	60.40 ± 2.68	47.76 ± 4.63
	SA-GEL		nd	nd	nd	95.57 ± 2.78	140.55 ± 2.35
Syringic acid	C_{CS}	88.88 ± 20.39	9.94 ± 0.04	-	3.10 ± 0.06	-	nd
	SA		5.53 ± 0.21	54.22 ± 3.98	22.42 ± 0.55	nd	nd
	SA-GA		nd	4.43 ± 0.51	nd	nd	nd
	SA-GEL		56.06 ± 0.48	21.74 ± 1.83	2.85 ± 0.22	nd	nd
Vanillic acid	C_{CS}	10.45 ± 0.80	nd	-	nd	-	2.39 ± 0.11
	SA		0.34 ± 0.00	4.16 ± 0.21	4.21 ± 0.42	4.54 ± 0.62	5.53 ± 0.07
	SA-GA		nd	nd	nd	7.93 ± 0.72	6.11 ± 0.21
	SA-GEL		8.54 ± 0.48	1.40 ± 0.07	nd	8.28 ± 0.59	9.15 ± 0.29
Ellagic acid	C_{CS}	94.72 ± 10.39	nd	-	nd	-	1.63 ± 0.11
	SA		nd	nd	nd	9.18 ± 0.14	8.52 ± 0.42
	SA-GA		nd	nd	nd	9.97 ± 1.13	14.18 ± 1.75
	SA-GEL		nd	nd	nd	16.05 ± 1.32	36.07 ± 0.15
p-Hydroxybenzoic acid	C_{CS}	2.97 ± 0.54	nd	-	nd	-	nd
	SA		nd	nd	nd	nd	nd
	SA-GA		nd	nd	nd	nd	nd
	SA-GEL		nd	nd	nd	nd	nd
o-Coumaric acid	C_{CS}	7.46 ± 1.39	nd	-	nd	-	5.87 ± 0.93
	SA		nd	nd	nd	27.58 ± 0.62	14.25 ± 1.18
	SA-GA		nd	nd	nd	24.09 ± 1.13	21.45 ± 2.78

Table S1. Release profile of individual phenolics from Cabernet Sauvignon control extract (C_{CS}) and from SA, SA-GA and SA-GEL microcapsules before digestion (BD) and during all phases of simulated in vitro digestion.

**Component**	**Sample**	**Before digestion**	**Oral phase**	**Gastric phase**		**Intestinal phase**	
		**BD**	**OP_3**	**GP_{63}**	**GP_{123}**	**IP_{183}**	**IP_{243}**
	SA-GEL		nd	nd	nd	34.38 ± 4.10	25.16 ± 2.35
p-Coumaric acid	C_{CS}	3.43 ± 0.71	0.59 ± 0.14	-	0.21 ± 0.07	-	2.99 ± 0.11
	SA		nd	nd	nd	nd	nd
	SA-GA		nd	nd	nd	nd	nd
	SA-GEL		nd	nd	nd	nd	nd
Caffeic acid	C_{CS}	1.40 ± 0.04	nd	-	nd	-	nd
	SA		nd	nd	nd	nd	nd
	SA-GA		nd	nd	nd	nd	nd
	SA-GEL		nd	nd	nd	nd	nd
Ferulic acid	C_{CS}	0.97 ± 0.01	nd	-	nd	-	nd
	SA		nd	nd	nd	nd	nd
	SA-GA		nd	nd	nd	nd	nd
	SA-GEL		nd	nd	nd	nd	nd
***Stilbenes* (µg/100 mg_{EXT})**							
Resveratrol	C_{CS}	7.91 ± 0.45	nd	-	nd	-	nd
	SA		nd	nd	nd	nd	nd
	SA-GA		nd	nd	nd	nd	nd
	SA-GEL		nd	nd	nd	nd	nd
ε-Viniferin	C_{CS}	22.75 ± 0.58	nd	-	nd	-	nd
	SA		nd	nd	nd	nd	nd
	SA-GA		nd	nd	nd	nd	nd
	SA-GEL		nd	nd	nd	nd	nd
***Flavanols* (µg/100 mg_{EXT})**							
Epicatechin	C_{CS}	100.71 ± 11.65	79.11 ± 1.67	-	34.62 ± 0.71	-	50.19 ± 1.13
	SA		18.76 ± 2.93	28.98 ± 1.66	29.20 ± 2.04	108.95 ± 5.25	57.58 ± 1.25
	SA-GA		8.09 ± 1.96	88.10 ± 3.29	70.15 ± 5.24	167.23 ± 7.62	98.22 ± 13.06
	SA-GEL		96.66 ± 1.73	49.39 ± 5.14	74.80 ± 1.61	245.61 ± 7.61	140.14 ± 1.76
Catechin	C_{CS}	240.87 ± 13.61	61.78 ± 1.82	-	12.14 ± 0.57	-	nd

gels

MDPI

Table S1. Release profile of individual phenolics from Cabernet Sauvignon control extract (C_{CS}) and from SA, SA-GA and SA-GEL microcapsules before digestion (BD) and during all phases of simulated in vitro digestion.

**Component**	**Sample**	**Before digestion**	**Oral phase**	**Gastric phase**		**Intestinal phase**	
		**BD**	**OP_3**	**GP_{63}**	**GP_{123}**	**IP_{183}**	**IP_{243}**
	SA		10.76 ± 0.09	13.15 ± 1.83	14.17 ± 0.87	nd	nd
	SA-GA		nd	78.87 ± 10.48	50.23 ± 9.66	nd	nd
	SA-GEL		52.97 ± 3.38	13.75 ± 2.13	36.00 ± 0.73	nd	nd
Epicatechin gallate	C_{CS}	5.78 ± 2.74	nd	-	6.99 ± 0.16	-	6.75 ± 0.76
	SA		nd	14.52 ± 0.38	9.79 ± 0.42	12.59 ± 0.42	6.56 ± 1.38
	SA-GA		nd	11.87 ± 0.56	11.89 ± 1.18	19.07 ± 2.47	10.76 ± 1.64
	SA-GEL		nd	nd	nd	22.26 ± 0.44	20.58 ± 0.59
Gallocatechin gallate	C_{CS}	72.53 ± 21.84	nd	-	nd	-	182.35 ± 7.51
	SA		nd	nd	nd	613.91 ± 3.24	502.21 ± 16.06
	SA-GA		nd	nd	nd	769.05 ± 4.73	711.28 ± 12.95
	SA-GEL		nd	nd	nd	1032.97 ± 7.03	1471.43 ± 10.88
Procyanidin B1	C_{CS}	118.52 ± 25.63	21.28 ± 1.36	-	nd	-	nd
	SA		nd	nd	nd	108.85 ± 13.94	84.36 ± 9.62
	SA-GA		nd	nd	nd	122.26 ± 5.15	154.48 ± 4.83
	SA-GEL		nd	nd	nd	160.08 ± 2.34	183.80 ± 7.35
Procyanidin B2	C_{CS}	46.57 ± 21.88	22.85 ± 0.34	-	8.81 ± 0.07	-	nd
	SA		7.03 ± 1.87	74.91 ± 4.50	13.29 ± 0.59	nd	nd
	SA-GA		nd	nd	nd	nd	nd
	SA-GEL		29.29 ± 0.04	22.83 ± 4.99	nd	nd	nd
***Flavonols* (μg/100 mg_{EXT})**							
Quercetin	C_{CS}	214.33 ± 24.44	17.75 ± 0.06	-	1.60 ± 0.08	-	nd
	SA		nd	nd	nd	nd	nd
	SA-GA		nd	nd	nd	nd	nd
	SA-GEL		nd	nd	nd	nd	nd
Rutin	C_{CS}	13.14 ± 3.60	4.95 ± 0.13	-	2.53 ± 0.07	-	nd
	SA		nd	nd	nd	nd	nd
	SA-GA		nd	nd	nd	nd	nd
	SA-GEL		nd	nd	nd	nd	nd

Table S1. Release profile of individual phenolics from Cabernet Sauvignon control extract (C_{CS}) and from SA, SA-GA and SA-GEL microcapsules before digestion (BD) and during all phases of simulated in vitro digestion.

Component	Sample	Before digestion	Oral phase	Gastric phase		Intestinal phase	
		**BD**	**OP_{3}**	**GP_{63}**	**GP_{123}**	**IP_{183}**	**IP_{243}**
Kaempferol	C_{CS}	13.22 ± 1.34	nd	-	nd	-	nd
	SA		nd	nd	nd	nd	nd
	SA-GA		nd	nd	nd	nd	nd
	SA-GEL		nd	nd	nd	nd	nd
***Anthocyanins* (µg/100 mg_{EXT})**							
Oenin chloride	C_{CS}	91.28 ± 0.72	47.12 ± 0.25	-	64.71 ± 0.01	-	31.42 ± 2.56
	SA		27.62 ± 1.02	94.56 ± 0.17	40.05 ± 0.14	42.61 ± 13.18	8.32 ± 1.25
	SA-GA		10.96 ± 0.10	98.08 ± 1.18	81.97 ± 0.46	38.28 ± 17.29	25.59 ± 3.29
	SA-GEL		141.33 ± 0.88	99.39 ± 4.55	94.73 ± 8.95	61.20 ± 8.64	58.32 ± 9.56
Myrtillin chloride	C_{CS}	12.09 ± 0.04	2.29 ± 0.03	-	nd	-	nd
	SA		nd	nd	nd	nd	nd
	SA-GA		nd	nd	nd	nd	nd
	SA-GEL		nd	nd	nd	nd	nd
Petunidin chloride	C_{CS}	1.89 ± 0.08	0.96 ± 0.04	-	nd	-	nd
	SA		nd	nd	nd	nd	nd
	SA-GA		nd	nd	nd	nd	nd
	SA-GEL		nd	nd	nd	nd	nd
Peonidin-3-*O*-glucoside chloride	C_{CS}	9.76 ± 0.02	5.40 ± 0.04	-	6.29 ± 0.21	-	2.41 ± 0.42
	SA		1.97 ± 0.16	6.49 ± 0.17	nd	nd	nd
	SA-GA		nd	6.97 ± 0.82	5.42 ± 0.05	nd	nd
	SA-GEL		11.36 ± 0.70	8.35 ± 0.66	9.03 ± 0.59	nd	nd
Kuromanin chloride	C_{CS}	2.29 ± 0.00	nd	-	nd	-	nd
	SA		nd	nd	nd	nd	nd
	SA-GA		nd	nd	nd	nd	nd
	SA-GEL		nd	nd	nd	nd	nd

GP – gastric phase, IP – intestinal phase, OP – oral phase, nd – not detected, ″-„ – not determined. Index numbers associated with abbreviations indicate the time interval when a certain sample was taken (i.e. .GP_{123} – 123rd minute of the gastric phase). For the C_{CS}, only the endpoints of the oral, gastric, and intestinal phases are shown (OP_{3}, GP_{123}, IP_{243}). Phenolic contents are expressed as mean value (µg/100 mg_{EXT}) ± SD.

gels

MDPI

Table S2. Release profile of individual phenolics from Cabernet Franc control extract (C_{CF}) and from SA, SA-GA and SA-GEL microcapsules before digestion (BD) and during all phases of simulated in vitro digestion.

Component	Sample	Before digestion	Oral phase	Gastric phase		Intestinal phase	
		BD	OP_3	GP_{63}	GP_{123}	IP_{183}	IP_{243}
***Phenolic acids* (μg/100 mg$_{EXT}$)**							
Gallic acid	C_{CF}	130.00 ± 10.98	2.09 ± 0.06	-	1.19 ± 0.17	-	141.38 ± 6.37
	SA		nd	1.74 ± 0.10	1.16 ± 0.03	23.87 ± 1.24	47.97 ± 0.52
	SA-GA		0.78 ± 0.01	nd	nd	52.46 ± 1.47	23.64 ± 0.00
	SA-GEL		3.01 ± 0.06	nd	nd	103.68 ± 0.85	124.35 ± 0.46
3,4-Dihydroxybenzoic acid	C_{CF}	24.09 ± 1.97	nd	-	nd	-	17.01 ± 0.08
	SA		nd	nd	nd	10.69 ± 0.66	16.16 ± 0.88
	SA-GA		nd	nd	nd	17.81 ± 0.04	17.89 ± 0.68
	SA-GEL		nd	nd	nd	32.13 ± 1.25	35.90 ± 0.91
Syringic acid	C_{CF}	114.36 ± 3.02	6.92 ± 0.78	-	nd	-	nd
	SA		nd	0.79 ± 0.25	3.06 ± 0.54	1.43 ± 0.16	3.09 ± 0.02
	SA-GA		nd	nd	nd	3.46 ± 0.30	1.78 ± 0.11
	SA-GEL		6.60 ± 0.99	nd	nd	nd	nd
Vanillic acid	C_{CF}	14.83 ± 0.74	nd	-	nd	-	4.39 ± 0.00
	SA		nd	2.41 ± 0.13	2.09 ± 0.10	1.25 ± 0.16	1.41 ± 0.13
	SA-GA		nd	nd	nd	2.85 ± 0.19	3.35 ± 0.08
	SA-GEL		nd	nd	nd	3.74 ± 0.06	3.56 ± 0.00
Ellagic acid	C_{CF}	81.33 ± 1.23	nd	-	nd	-	1.28 ± 0.00
	SA		nd	0.45 ± 0.07	0.56 ± 0.00	1.24 ± 0.07	nd
	SA-GA		nd	nd	nd	2.82 ± 0.45	6.39 ± 0.15
	SA-GEL		nd	nd	nd	5.15 ± 0.34	6.87 ± 0.34
p-Hydroxybenzoic acid	C_{CF}	1.00 ± 0.04	nd	-	nd	-	nd
	SA		nd	nd	nd	nd	nd
	SA-GA		nd	nd	nd	nd	nd
	SA-GEL		nd	nd	nd	nd	nd
o-Coumaric acid	C_{CF}	19.48 ± 0.58	nd	-	nd	-	9.37 ± 0.06
	SA		nd	nd	nd	7.16 ± 0.13	12.43 ± 0.58
	SA-GA		nd	nd	nd	6.84 ± 0.64	3.81 ± 0.34
	SA-GEL		nd	nd	nd	7.57 ± 0.46	10.67 ± 0.34
p-Coumaric acid	C_{CF}	1.44 ± 0.04	0.43 ± 0.06	-	0.44 ± 0.00	-	3.93 ± 0.70
	SA		nd	nd	nd	nd	nd
	SA-GA		nd	nd	nd	nd	nd

Component	Sample	Before digestion	Oral phase	Gastric phase		Intestinal phase	
		BD	OP_3	GP_{63}	GP_{123}	IP_{183}	IP_{243}
	SA-GEL		nd	nd	nd	nd	nd
Caffeic acid	C_{CF}	1.04 ± 0.02	nd	-	nd	-	nd
	SA		nd	nd	nd	nd	nd
	SA-GA		nd	nd	nd	nd	nd
	SA-GEL		nd	nd	nd	nd	nd
Ferulic acid	C_{CF}	2.52 ± 0.25	nd	-	nd	-	nd
	SA		nd	nd	nd	nd	nd
	SA-GA		nd	nd	nd	nd	nd
	SA-GEL		nd	nd	nd	nd	nd
Stilbenes (μg/100 mg_{EXT})							
Resveratrol	C_{CF}	8.82 ± 0.40	nd	-	nd	-	nd
	SA		nd	nd	nd	nd	nd
	SA-GA		nd	nd	nd	nd	nd
	SA-GEL		nd	nd	nd	nd	nd
ε-Viniferin	C_{CF}	13.72 ± 0.06	nd	-	nd	-	nd
	SA		nd	nd	nd	nd	nd
	SA-GA		nd	nd	nd	nd	nd
	SA-GEL		nd	nd	nd	nd	nd
Flavanols (μg/100 mg_{EXT})							
Epicatechin	C_{CF}	547.27 ± 25.23	343.19 ± 7.06	-	324.16 ± 4.16	-	nd
	SA		nd	nd	nd	28.38 ± 1.17	19.62 ± 0.67
	SA-GA		nd	76.58 ± 0.79	49.87 ± 1.40	23.72 ± 2.67	26.83 ± 4.37
	SA-GEL		nd	nd	nd	78.31 ± 0.51	48.23 ± 2.00
Catechin	C_{CF}	527.59 ± 28.62	100.95 ± 12.01	-	139.84 ± 2.90	-	nd
	SA		3.41 ± 0.21	nd	nd	nd	nd
	SA-GA		nd	46.33 ± 0.09	24.80 ± 0.57	nd	nd
	SA-GEL		47.62 ± 3.86	nd	nd	nd	nd
Epicatechin gallate	C_{CF}	32.46 ± 1.65	nd	-	nd	-	32.06 ± 0.06
	SA		nd	2.88 ± 0.46	1.53 ± 0.02	2.24 ± 0.23	1.89 ± 0.09
	SA-GA		nd	4.02 ± 0.11	4.25 ± 0.51	4.02 ± 0.04	11.92 ± 1.20
	SA-GEL		nd	nd	nd	7.05 ± 0.74	6.35 ± 0.51
Gallocatechin gallate	C_{CF}	127.23 ± 9.54	nd	-	nd	-	190.83 ± 6.03
	SA		nd	nd	nd	131.50 ± 0.93	153.00 ± 0.61
	SA-GA		nd	nd	nd	288.51 ± 4.03	251.35 ± 11.10

gels

MDPI

Component	Sample	Before digestion	Oral phase	Gastric phase		Intestinal phase	
		BD	OP_3	GP_{63}	GP_{123}	IP_{183}	IP_{243}
	SA-GEL		nd	nd	nd	451.57 ± 5.75	443.02 ± 1.09
Procyanidin B1	C_{CF}	317.42 ± 2.59	60.20 ± 0.10	-	72.36 ± 7.13	-	nd
	SA		nd	nd	nd	nd	nd
	SA-GA		nd	nd	nd	nd	nd
	SA-GEL		nd	nd	nd	nd	nd
Procyanidin B2	C_{CF}	126.51 ± 23.32	111.71 ± 5.15	-	137.25 ± 3.75	-	nd
	SA		nd	138.25 ± 10.40	124.86 ± 0.68	6.62 ± 0.18	14.04 ± 1.94
	SA-GA		nd	20.43 ± 2.05	13.28 ± 1.77	8.30 ± 0.15	12.24 ± 0.15
	SA-GEL		241.83 ± 5.52	nd	nd	nd	nd
Flavonols **(µg/100 mg_{EXT})**							
Quercetin	C_{CF}	146.04 ± 3.93	17.75 ± 0.06	-	1.60 ± 0.08	-	nd
	SA		nd	nd	nd	nd	nd
	SA-GA		nd	nd	nd	nd	nd
	SA-GEL		nd	nd	nd	nd	nd
Rutin	C_{CF}	65.08 ± 5.10	4.95 ± 0.13	-	2.53 ± 0.07	-	nd
	SA		nd	nd	nd	nd	nd
	SA-GA		nd	nd	nd	nd	nd
	SA-GEL		nd	nd	nd	nd	nd
Kaempferol	C_{CF}	10.40 ± 1.00	nd	-	nd	-	nd
	SA		nd	nd	nd	nd	nd
	SA-GA		nd	nd	nd	nd	nd
	SA-GEL		nd	nd	nd	nd	nd
Anthocyanins **(µg/100 mg_{EXT})**							
Oenin chloride	C_{CF}	511.54 ± 1.17	-	733.77 ± 1.11	-	259.78 ± 23.24	511.54 ± 1.17
	SA	42.58 ± 0.14	54.90 ± 0.13	45.87 ± 0.44	41.86 ± 2.19	37.16 ± 0.49	42.58 ± 0.14
	SA-GA	27.56 ± 0.54	275.81 ± 0.23	209.88 ± 0.36	91.08 ± 6.32	26.96 ± 2.60	27.56 ± 0.54
	SA-GEL	188.30 ± 0.17	223.21 ± 1.85	201.89 ± 0.11	121.72 ± 8.14	80.89 ± 5.77	188.30 ± 0.17
Myrtillin chloride	C_{CF}	7.98 ± 0.30	-	6.73 ± 0.01	-	nd	7.98 ± 0.30
	SA	nd	nd	nd	nd	nd	nd
	SA-GA	nd	nd	nd	nd	nd	nd
	SA-GEL	nd	nd	nd	nd	nd	nd
Petunidin chloride	C_{CF}	3.11 ± 0.17	-	3.62 ± 0.22	-	nd	3.11 ± 0.17
	SA	nd	nd	nd	nd	nd	nd
	SA-GA	nd	nd	nd	nd	nd	nd

gels

MDPI

Component	Sample	Before digestion	Oral phase	Gastric phase		Intestinal phase	
		BD	OP_3	GP_{63}	GP_{123}	IP_{183}	IP_{243}
	SA-GEL	nd	nd	nd	nd	nd	nd
Peonidin-3-*O*-glucoside chloride	C_{CF}	45.42 ± 0.90	-	61.59 ± 1.32	-	18.64 ± 2.23	45.42 ± 0.90
	SA	2.58 ± 0.10	4.17 ± 0.49	3.18 ± 0.13	2.88 ± 0.30	1.96 ± 0.25	2.58 ± 0.10
	SA-GA	1.35 ± 0.20	20.46 ± 2.05	13.28 ± 0.15	4.87 ± 1.02	nd	1.35 ± 0.20
	SA-GEL	14.89 ± 0.30	13.74 ± 0.66	11.92 ± 0.20	8.33 ± 1.54	nd	14.89 ± 0.30
Kuromanin chloride	C_{CF}	nd	-	nd	-	nd	nd
	SA	nd	nd	nd	nd	nd	nd
	SA-GA	nd	nd	nd	nd	nd	nd
	SA-GEL	nd	nd	nd	nd	nd	nd

GP – gastric phase, IP – intestinal phase, OP – oral phase, nd – not detected, ″-„ – not determined. Index numbers associated with abbreviations indicate the time interval when a certain sample was taken (i.e. GP_{123} – 123rd minute of the gastric phase). For the C_{CF}, only the endpoints of the oral, gastric, and intestinal phases are shown (OP_3, GP_{123}, IP_{243}). Phenolic contents are expressed as mean value (µg/100 mg_{EXT}) ± SD.

gels

MDPI

Table S3. Release profile of individual phenolics from Merlot control extract (C_M) and from SA, SA-GA and SA-GEL microcapsules before digestion (BD) and during all phases of simulated in vitro digestion.

Component	Sample	Before digestion	Oral phase	Gastric phase		Intestinal phase	
		BD	OP_3	GP_{63}	GP_{123}	IP_{183}	IP_{243}
***Phenolic acids* (μg/100 mg$_{EXT}$)**							
Gallic acid	C_M	207.79 ± 10.15	1.57 ± 0.16	-	1.61 ± 0.04	-	98.41 ± 0.59
	SA		0.38 ± 0.06	14.91 ± 0.72	18.57 ± 0.03	138.18 ± 2.44	55.88 ± 1.56
	SA-GA		0.89 ± 0.13	1.78 ± 0.00	1.42 ± 0.38	207.12 ± 10.68	462.43 ± 15.11
	SA-GEL		1.60 ± 0.00	3.79 ± 0.09	3.07 ± 0.94	399.78 ± 1.13	454.06 ± 57.52
3,4-Dihydroxybenzoic acid	C_M	75.63 ± 5.51	nd	-	nd	-	9.33 ± 0.17
	SA		nd	14.00 ± 0.06	17.95 ± 0.16	42.00 ± 0.25	50.60 ± 0.93
	SA-GA		nd	nd	nd	104.40 ± 3.39	119.30 ± 2.01
	SA-GEL		nd	nd	nd	158.34 ± 6.24	179.09 ± 4.34
Syringic acid	C_M	51.89 ± 9.07	12.14 ± 2.90	-	11.88 ± 0.13	-	nd
	SA		nd	nd	nd	7.80 ± 0.00	3.52 ± 0.12
	SA-GA		nd	nd	nd	nd	48.79 ± 0.76
	SA-GEL		nd	nd	nd	13.76 ± 0.19	14.54 ± 2.83
Vanillic acid	C_M	12.34 ± 0.25	nd	-	nd	-	2.72 ± 0.08
	SA		nd	2.99 ± 0.22	4.56 ± 0.06	3.77 ± 0.06	6.08 ± 0.00
	SA-GA		nd	nd	nd	9.33 ± 0.88	17.81 ± 0.50
	SA-GEL		nd	nd	nd	15.90 ± 0.19	8.93 ± 0.57
Ellagic acid	C_M	16.32 ± 0.12	nd	-	nd	-	2.52 ± 0.08
	SA		nd	nd	nd	5.09 ± 0.19	11.45 ± 0.37
	SA-GA		nd	nd	nd	5.69 ± 0.75	14.78 ± 1.26
	SA-GEL		nd	nd	nd	8.69 ± 0.94	17.60 ± 3.02
p-Hydroxybenzoic acid	C_M	2.10 ± 0.09	nd	-	nd	-	nd
	SA		nd	nd	nd	nd	nd
	SA-GA		nd	nd	nd	nd	nd
	SA-GEL		nd	nd	nd	nd	nd
o-Coumaric acid	C_M	9.60 ± 0.60	nd	-	nd	-	5.92 ± 0.06
	SA		nd	nd	nd	45.82 ± 0.19	5.55 ± 1.37
	SA-GA		nd	nd	nd	23.37 ± 0.38	31.25 ± 0.63
	SA-GEL		nd	nd	nd	38.35 ± 3.21	44.54 ± 6.41
p-Coumaric acid	C_M	3.31 ± 0.20	0.28 ± 0.03	-	0.36 ± 0.03	-	2.16 ± 0.08
	SA		nd	nd	nd	nd	nd
	SA-GA		nd	nd	nd	nd	nd

Component	Sample	Before digestion	Oral phase	Gastric phase		Intestinal phase	
		BD	OP_3	GP_{63}	GP_{123}	IP_{183}	IP_{243}
	SA-GEL		nd	nd	nd	nd	nd
Caffeic acid	C_M	0.75 ± 0.01	nd	-	nd	-	nd
	SA		nd	nd	nd	nd	nd
	SA-GA		nd	nd	nd	nd	nd
	SA-GEL		nd	nd	nd	nd	nd
Ferulic acid	C_M	0.97 ± 0.08	nd	-	nd	-	nd
	SA		nd	nd	nd	nd	nd
	SA-GA		nd	nd	nd	nd	nd
	SA-GEL		nd	nd	nd	nd	nd
***Stilbenes* (µg/100 mg_{EXT})**							
Resveratrol	C_M	14.16 ± 0.37	nd	-	nd	-	nd
	SA		nd	nd	nd	nd	nd
	SA-GA		nd	nd	nd	nd	nd
	SA-GEL		nd	nd	nd	nd	nd
ε-Viniferin	C_M	8.53 ± 0.31	nd	-	nd	-	nd
	SA		nd	nd	nd	nd	nd
	SA-GA		nd	nd	nd	nd	nd
	SA-GEL		nd	nd	nd	nd	nd
***Flavanols* (µg/100 mg_{EXT})**							
Epicatechin	C_M	248.31 ± 9.17	190.58 ± 9.18	-	200.57 ± 2.08	-	38.79 ± 0.53
	SA		25.46 ± 4.56	16.39 ± 1.25	17.88 ± 2.82	75.58 ± 4.39	nd
	SA-GA		nd	44.47 ± 0.44	41.68 ± 6.20	151.67 ± 5.40	132.83 ± 1.76
	SA-GEL		19.36 ± 2.08	162.90 ± 16.94	220.09 ± 20.08	294.22 ± 11.72	207.49 ± 11.69
Catechin	C_M	272.26 ± 0.42	101.90 ± 6.52	-	98.69 ± 1.03	-	nd
	SA		9.41 ± 0.61	20.67 ± 3.29	38.93 ± 2.47	nd	nd
	SA-GA		nd	nd	nd	nd	nd
	SA-GEL		nd	85.11 ± 4.99	103.28 ± 4.81	nd	nd
Epicatechin gallate	C_M	8.29 ± 0.04	nd	-	nd	-	7.35 ± 0.73
	SA		nd	8.88 ± 1.28	11.15 ± 0.25	6.73 ± 0.00	30.47 ± 3.74
	SA-GA		nd	5.88 ± 0.63	9.35 ± 0.19	29.77 ± 0.13	38.19 ± 2.14
	SA-GEL		nd	15.97 ± 1.32	24.54 ± 3.96	36.88 ± 0.76	39.47 ± 5.66
Gallocatechin gallate	C_M	76.11 ± 4.85	104.18 ± 3.47	-	114.91 ± 2.29	-	178.11 ± 0.17
	SA		nd	nd	nd	447.37 ± 22.18	441.97 ± 13.20
	SA-GA		nd	nd	nd	787.15 ± 8.92	1021.90 ± 7.81

Component	Sample	Before digestion	Oral phase	Gastric phase		Intestinal phase	
		BD	OP_3	GP_{63}	GP_{123}	IP_{183}	IP_{243}
	SA-GEL		nd	nd	nd	1298.22 ± 24.19	1500.06 ± 1.32
Procyanidin B1	C_M	197.93 ± 16.51	28.71 ± 0.46	-	44.79 ± 5.83	-	nd
	SA		nd	nd	nd	109.03 ± 1.69	95.78 ± 8.91
	SA-GA		nd	nd	nd	177.08 ± 17.72	235.66 ± 13.98
	SA-GEL		nd	nd	nd	250.13 ± 1.51	268.03 ± 29.42
Procyanidin B2	C_M	62.19 ± 2.76	58.30 ± 2.27	-	61.28 ± 0.54	-	nd
	SA		nd	74.85 ± 1.72	148.35 ± 46.00	nd	nd
	SA-GA		nd	nd	nd	nd	176.46 ± 6.55
	SA-GEL		nd	nd	nd	nd	nd
Flavonols (µg/100 mg_{EXT})							
Quercetin	C_M	120.95 ± 1.84	13.12 ± 0.81	-	5.93 ± 0.31	-	nd
	SA		nd	nd	nd	nd	nd
	SA-GA		nd	nd	nd	nd	nd
	SA-GEL		nd	nd	nd	nd	nd
Rutin	C_M	24.13 ± 0.95	7.00 ± 1.00	-	8.30 ± 0.01	-	nd
	SA		nd	nd	nd	nd	nd
	SA-GA		nd	nd	nd	nd	nd
	SA-GEL		nd	nd	nd	nd	nd
Kaempferol	C_M	5.19 ± 0.06	nd	-	nd	-	nd
	SA		nd	nd	nd	nd	nd
	SA-GA		nd	nd	nd	nd	nd
	SA-GEL		nd	nd	nd	nd	nd
Anthocyanins (µg/100 mg_{EXT})							
Oenin chloride	C_M	32.83 ± 0.36	26.58 ± 0.64	-	38.08 ± 0.28	-	20.56 ± 1.49
	SA		7.84 ± 0.17	12.83 ± 0.16	12.97 ± 1.00	13.02 ± 1.13	4.89 ± 0.06
	SA-GA		3.27 ± 0.66	12.73 ± 0.76	14.57 ± 0.69	13.77 ± 0.13	13.18 ± 0.00
	SA-GEL		22.59 ± 0.42	28.55 ± 0.09	41.21 ± 4.15	18.71 ± 0.76	14.67 ± 1.51
Myrtillin chloride	C_M	2.81 ± 0.01	nd	-	nd	-	nd
	SA		nd	nd	nd	nd	nd
	SA-GA		nd	nd	nd	nd	nd
	SA-GEL		nd	nd	nd	nd	nd
Petunidin chloride	C_M	0.67 ± 0.03	0.27 ± 0.01	-	nd	-	nd
	SA		nd	nd	nd	nd	nd
	SA-GA		nd	nd	nd	nd	nd

Component	Sample	Before digestion	Oral phase	Gastric phase		Intestinal phase	
		BD	OP_3	GP_{63}	GP_{123}	IP_{183}	IP_{243}
	SA-GEL		nd	nd	nd	nd	nd
Peonidin-3-*O*-glucoside chloride	C_M	7.17 ± 0.09	5.49 ± 0.20	-	7.34 ± 0.13	-	3.04 ± 0.42
	SA		0.92 ± 0.08	nd	nd	nd	nd
	SA-GA		nd	2.72 ± 0.31	1.86 ± 0.25	nd	nd
	SA-GEL		3.67 ± 0.47	5.12 ± 0.28	4.60 ± 1.60	nd	nd
Kuromanin chloride	C_M	1.11 ± 0.15	nd	-	nd	-	nd
	SA		nd	nd	nd	nd	nd
	SA-GA		nd	nd	nd	nd	nd
	SA-GEL		nd	nd	nd	nd	nd

GP – gastric phase, IP – intestinal phase, OP – oral phase, nd – not detected, ˝-„ – not determined. Index numbers associated with abbreviations indicate the time interval when a certain sample was taken (i.e. GP_{123} – 123rd minute of the gastric phase). For the C_M, only the endpoints of the oral, gastric, and intestinal phases are shown (OP_3, GP_{123}, IP_{243}). Phenolic contents are expressed as mean value (μg/100 mg_{EXT}) ± SD.
